# Deletions of recombination genes impair tandem amplification and reshape heteroresistance mechanisms in *Escherichia coli*

**DOI:** 10.1128/mbio.03674-25

**Published:** 2026-01-12

**Authors:** Sheida Heidarian, Karin Hjort, Hervé Nicoloff, Dan I. Andersson

**Affiliations:** 1 Department of Medical Biochemistry and Microbiology, Uppsala University 8097, Uppsala, Sweden; Universite de Geneve, Geneva, Switzerland

**Keywords:** heteroresistance, DNA recombination, gene amplification, *Escherichia coli*, resistance gene

## Abstract

Heteroresistance is a transient resistance phenotype characterized by the presence of small subpopulations of bacterial cells with elevated antibiotic resistance within a susceptible main population. In gram-negative pathogens, heteroresistance is frequently caused by tandem amplification of genes encoding resistance proteins with low activity toward the antibiotic, a process commonly mediated by homologous recombination between flanking repeated sequences. However, the specific roles of individual recombination proteins in this mechanism remain largely undefined. In this study, we systematically evaluated the contribution of 19 recombination-associated genes to tandem amplification-driven heteroresistance in *Escherichia coli*. A clinical plasmid causing tobramycin heteroresistance by tandem amplification of the *aac(3)-IId* gene was conjugated into recombination gene-deficient mutants and the wild-type parental strain. While heteroresistance was observed with all mutants, the frequency of resistant subpopulations was decreased in *recA* and *recB* mutants, and a shift in resistance mechanism toward increased plasmid copy number and resistance mutations was observed. Partially reduced frequencies of tandem amplifications and a shift toward other heteroresistance mechanisms were also observed with *recC*, *recJ, ruvA*, and *ruvC* mutants, whereas other deletions of recombination genes had no or little impact on tandem amplifications. These findings identify RecABC as a key pathway in heteroresistance *via* tandem amplification, but even when these genes are deleted, resistant subpopulations can still be generated by other mechanisms.

**IMPORTANCE:**

Heteroresistance poses a threat to efficient antibiotic treatment, as the rare resistant subpopulations often go undetected by standard laboratory tests. In *Escherichia coli*, heteroresistance often arises by tandem gene amplification of a resistance gene with low activity toward the specific antibiotic. This amplification is thought to be mediated by homologous recombination between repeat sequences. However, the specific roles of individual recombination proteins in this process are unclear. Here, we systematically determined the specific roles of individual recombination proteins in this process by the individual deletion of 19 recombination-associated genes. The RecABC pathway was identified as a major contributor to amplification-driven heteroresistance, but even when this pathway was disrupted, resistant subpopulations still emerged through alternative mechanisms, revealing the remarkable adaptability of bacterial populations under antibiotic stress. These findings advance our understanding of the molecular flexibility underlying heteroresistance and highlight that strategies aimed at preventing gene amplification to reduce heteroresistance are unlikely to succeed.

## INTRODUCTION

Heteroresistance (HR) is an antibiotic resistance phenotype characterized by the presence of a minor resistant subpopulation (typically ≈10^−7^ to 10^−4^ frequency) within a susceptible main population (1). This phenotype poses major challenges in the management of infectious diseases, as the resistant subpopulations, due to their low frequency, often are undetectable by conventional antimicrobial susceptibility tests, resulting in misclassification as susceptible (2, 3).

HR has been identified in numerous bacterial species, including both gram-positive and gram-negative species, against most classes of antibiotics (4–11). In recent years, HR research has intensified, as clinical evidence has highlighted the association between HR and treatment failure (9, 12–15). Importantly, HR subpopulations can evolve into stable resistant strains during antibiotic exposure, thereby potentially accelerating the development and dissemination of resistance (1, 16).

To date, two primary mechanisms have been shown to confer HR: (i) genetic alterations such as point mutations, insertions, deletions, or transposition of resistance genes into highly expressed ribosomal genes, and (ii) increased gene dosage of resistance genes with low activity toward the antibiotic when present at a single copy (1). The latter can occur through several processes, including tandem gene amplification, elevated plasmid copy number (PCN), and by transposition of resistance genes into small plasmids that can increase in copy number under antibiotic selection pressure (17, 18). Among these, tandem gene amplification, which typically arises via homologous recombination, has emerged as a predominant cause of HR in gram-negative bacteria (11). Homologous recombination plays a central role in DNA damage repair (19), shapes the evolution of bacterial genomes (20), and facilitates gene duplication (21, 22). Gene duplication often involves identical or highly similar sequences such as direct repeats (rRNA operons, IS elements, transposases) that are present at the border of the amplified unit. Unequal crossing over between these direct repeats present on sister chromatids during DNA replication is a major mechanism that can lead to the formation of tandem arrays of amplified DNA units (22).

The central mediator for homology searching within sequences in bacteria is RecA, a strand exchange protein. RecA is delivered to single-strand DNA (ssDNA) through one of two pathways: RecBCD (23) or RecFOR (24). Both pathways work to provide a ssDNA molecule coated with RecA to allow the invasion of a homologous molecule and continuity of the recombination process. In addition to the Rec family proteins (25), a wide range of other proteins, including members of the RuvABC resolvasome complex (branch migration and resolution of Holliday junctions) (26, 27) and the SbcBCD exonuclease complex (resolves DNA secondary structures like hairpins) (28), are also contributing to homologous recombination and play critical roles in maintaining genome stability and ensuring the fidelity of DNA repair and the recombination process (29, 30). RecA’s function in the SOS response (31–33), its potential to enhance the efficacy of various antibiotics upon inactivation (34–36), and its role in the evolution of antibiotic resistance (37) have been extensively studied. However, RecA’s contribution, alongside other recombination-associated proteins, to antibiotic resistance, particularly in the context of HR driven by tandem gene amplification, remains poorly understood and represents a critical gap in our current understanding of HR. To date, only two studies have directly investigated the role of recombination factors in the HR phenotype. One study demonstrated that inactivation of RecA partially or fully reverted HR in clinical *E. coli* isolates exposed to quinolones or β-lactams, primarily through suppression of tandem gene amplification of resistance determinants (38). In the second study, RecA inactivation completely disrupted the HR mechanism driven by tandem amplification of the *aadB* gene (39). However, the role of other recombination proteins involved in homologous recombination, which may also facilitate tandem gene amplification, remains unexplored. Given the central role of homologous recombination in generating tandem gene arrays, it is plausible that multiple proteins involved in recombination and DNA replication influence the emergence of HR phenotypes driven by gene amplification.

In this work, we evaluated the role of 19 recombination gene deletions in *E. coli* strains from the Keio collection using plasmid pDA33135-139, derived from a clinical tobramycin (TOB) HR isolate. This plasmid carries the aminoglycoside resistance gene *aac(3)-IId*, which undergoes tandem amplification via unequal crossing-over between flanking IS6 family transposase genes, thereby conferring a TOB HR phenotype. We demonstrated that deletions of *recA*, *recB*, *recC*, *recJ*, *ruvA,* and *ruvC* disrupted the tandem amplification of the target resistance gene. Nevertheless, HR was still observed in these recombination gene-deficient mutants, as the HR mechanism shifted predominantly from tandem amplification to increased PCN. This shift was particularly evident in *recA*- and *recB*-deficient mutants, where resistant subpopulations emerged exclusively through elevated PCN when selected at TOB concentrations exceeding the minimum inhibitory concentration (MIC) of the parental strain.

## MATERIALS AND METHODS

### Bacteria, media, and antibiotics

Twenty *E. coli* K-12 strains from the Keio collection, each carrying a single-gene deletion in a recombination-associated protein, were selected from our in-house bacterial strain collection (see Table 1 for the function of individual proteins) (40). A detailed list of these mutants is provided in Table S1. Unless otherwise stated, all experiments were performed using Mueller-Hinton (MH) broth and agar (Difco, Becton Dickinson Company). Luria-Bertani (LB) broth and agar (Sigma-Aldrich) were used exclusively during the construction of recombination gene-deficient mutants. Antibiotics were purchased from Sigma-Aldrich, and fresh stock solutions and supplemented agar plates were prepared fresh for each experiment to ensure optimal activity and consistency.

**TABLE 1:** List of genes encoding recombination functions and their role in recombination

Gene	Role in recombination
*recA*	Central protein that facilitates homologous pairing and strand exchange; essential for synapse formation
*recBC*	Multifunctional enzyme complex with helicase and nuclease activities; processes double-strand breaks and loads RecA at χ sites to initiate recombination
*recD*	Subunit of RecBCD complex; helicase, enhances exonuclease activity and influences the directionality of DNA degradation
*recF*	Part of the RecFOR complex; binds to single-stranded DNA and ATP, promoting RecA loading at single-strand gaps
*recJ*	5′→3′ single-stranded DNA exonuclease; processes DNA ends to generate substrates for RecA filament formation
*recN*	Structural maintenance protein involved in DNA damage response; promotes sister chromatid cohesion and RecA loading during double-strand break repair
*recO*	Facilitates annealing of complementary single-stranded DNA and assists RecA loading in the RecF pathway
*recQ*	Helicase that unwinds DNA structures at single-strand gaps; works with RecJ to prepare DNA for recombination
*recR*	Works with RecF and RecO to stabilize RecA filaments; binds double-stranded DNA and helps coordinate RecA loading
*recG*	Branch-specific helicase that promotes branch migration and resolution of recombination intermediates like Holliday junctions
*ruvA*	Binds Holliday junctions and recruits RuvB; initiates branch migration during recombination
*ruvB*	ATP-dependent helicase that drives branch migration of Holliday junctions in concert with RuvA
*ruvC*	Endonuclease that resolves Holliday junctions by cleaving DNA strands to complete recombination
*sbcB*	Exodeoxyribonuclease I that removes 3′ single-stranded DNA tails; facilitates RecBCD loading and recombination initiation
*sbcC*	Structure-specific nuclease that works with SbcD to process abnormal DNA structures and maintain genome stability
*sbcD*	Endonuclease/exonuclease that cleaves hairpins and other secondary DNA structures to initiate repair and recombination

All bacterial cultures were incubated at 37°C unless otherwise specified. Liquid cultures were grown under continuous agitation at 190 rpm, unless alternative conditions are specified in the relevant experimental sections.

### Construction of recombination gene-deficient mutants carrying plasmid pDA33135-139

#### Kanamycin cassette excision from Keio knockout strains using plasmid pCP20

To generate a clean genetic background free of antibiotic resistance markers, *E. coli* Keio knockout strains were cured of their kanamycin resistance cassette via FLP-mediated recombination using the temperature-sensitive plasmid pCP20 (41, 42). Cultures of individual mutants grown in low-salt LB medium with kanamycin (25 mg/L) at 37 °C were diluted 1:100 into fresh LB and incubated until OD₆₀₀ reached 0.2–0.4. Cells were placed on ice, harvested (4,500 × *g*, 7 min, 4°C), washed twice with sterile ice-cold 10% glycerol, and resuspended in 200 μL of 10% glycerol to prepare electrocompetent cells. Transformation was performed by electroporating 40 μL of competent cells with 2 μL of purified pCP20 plasmid DNA (isolated from *E. coli* DA24990 using the Plasmid DNA Mini Kit I, Omega) at 2.5 kV, 25 μF, and 200 Ω in a pre-chilled 0.2 cm cuvette. Immediately post-electroporation, 500 μL of pre-warmed SOC medium (20 g/L tryptone, 5 g/L yeast extract, 0.58 g/L NaCl, 0.186 g/L KCl, 20 mM MgSO4, and 20 mM glucose) was added, and cells were incubated at 30°C for 1 h with shaking. Transformants were selected at 30°C on LB agar plates containing ampicillin (50 mg/L) and re-streaked on the same medium to confirm plasmid acquisition. To excise the kanamycin cassette and eliminate pCP20, single colonies from LB agar plates containing ampicillin (50 mg/L) were incubated at 42°C for 24 h. Colonies were then patched onto LB agar plates supplemented or not with ampicillin (50 mg/L) or kanamycin (20 mg/L) to confirm loss of pCP20 and the resistance cassette, respectively. Clones that grew only on LB were selected as kanamycin cassette-free strains and stored at −80°C in LB supplemented with 10% DMSO.

#### Two-step conjugation for plasmid pDA33135-139 transfer into Keio knockout collection

**Fig 1:**
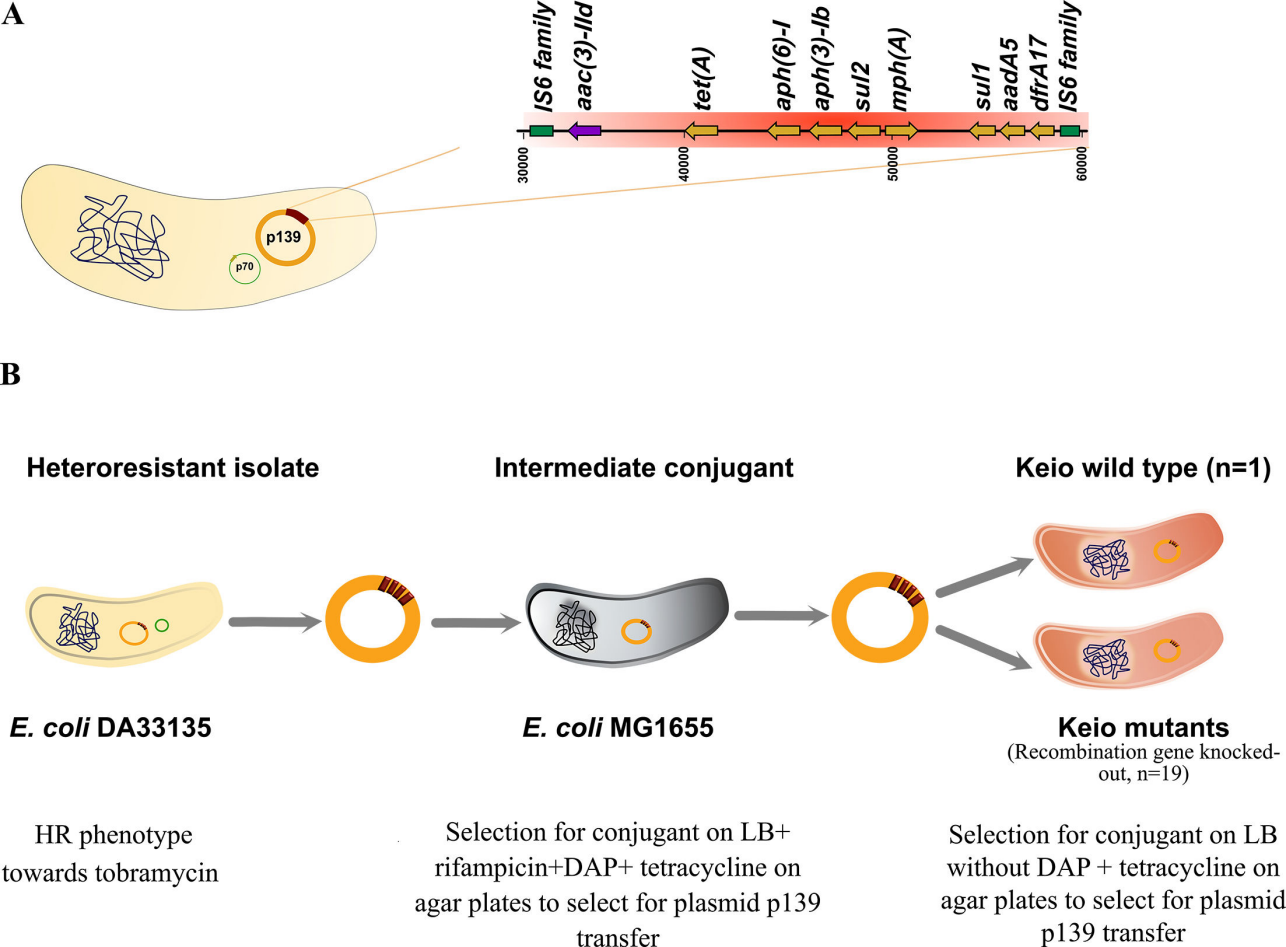
Experimental design for plasmid transfer and characterization of genetic content. (**A**) Illustration of *Escherichia coli* DA33135 and a close-up of a region of plasmid pDA33135-139 (p139). The highlighted region in red is the predicted amplified unit bordered by large identical sequences (IS6 family transposase). The *aac(3)-IId* gene is depicted in purple. (**B**) Conjugation-based strategy for transferring plasmid p139 from the TOB heteroresistant *E. coli* isolate DA33135 into individual Keio knockout strains.

To introduce the plasmid pDA33135-139 carrying the HR determinant (Fig. 1A) from the parental *E. coli* heteroresistant isolate (DA33135) into a panel of 20 individual Keio knockout mutants (kanamycin cassette-flipped) and the Keio wild-type strain, a two-step conjugation strategy was performed. A schematic overview of the procedure is provided in Fig. 1B.

Step 1: Conjugation of plasmid from DA33135 into intermediate 1655 strain. The donor strain (*E. coli* DA33135) harboring plasmid pDA33135-139 was cultured overnight in LB at 37°C. The intermediate recipient strain (*E. coli* MG1655), carrying rifampicin resistance and a diaminopimelic acid (DAP) auxotrophy, was grown under identical conditions in LB supplemented with 20 mg/L DAP. Equal volumes (300 μL) of each overnight culture were mixed, pelleted (5,000 × *g*, 2 min), and resuspended in 100 μL LB containing DAP (20 mg/L). The mixture was spotted onto a 0.45 μm nitrocellulose membrane (Whatman Protran BA 85, Ø 25 mm) placed on LB agar plate supplemented with DAP and incubated at 37°C for 20 h. Following incubation, cells were recovered by vortexing the membrane in 500 μL phosphate-buffered saline. Serial dilutions (undiluted and 1:10) were plated on LB agar containing rifampicin (100 μg/mL), tetracycline (15 μg/mL, resistance encoded on pDA33135-139), and DAP (20 μg/mL) to select for transconjugants. After 24 h at 37°C, colonies were screened by colony PCR using primers specific to a gene located on plasmid pDA33135-139 (*aph (6)-1d*) and a second primer set targeting a gene on plasmid pDA33135-70 (*bla*_CTX-M_) (Table S2). This dual screening ensured exclusive transfer of plasmid pDA33135-139 to the recipient cells.

Step 2: Conjugation of plasmid from intermediate transconjugant into Keio knockout strains. The intermediate strain *E. coli* MG1655 (rifampicin-resistant and DAP-auxotrophic) carrying plasmid pDA33135-139 was used as donor for conjugations into Keio knockout strains (devoid of the kanamycin resistance marker) and the Keio wild-type strain. The donor strain was cultured overnight in LB supplemented with 20 mg/L DAP, while recipients were grown in LB. Conjugation was performed as described in Step 1. Following conjugation and recovery, 100 μL of resuspended cells were plated on LB agar containing tetracycline (15 mg/L) and lacking DAP to select correct conjugants. Candidate conjugants were screened by colony PCR using two primer sets targeting *tet*(A) and *aac(3)-IId*, both present on pDA33135-139 (Table S2).

#### Validation of final Keio mutants carrying plasmid pDA33135-139

To confirm that plasmid acquisition (pDA33135-139) by the recombination gene deletion mutants did not result in altered gene dosage, all verified transconjugants were analyzed using droplet digital PCR (ddPCR, protocol described in a separate section). Gene copy number (GCN) of genes encoded on pDA33135-139 was compared between each Keio construct and the original parental HR isolate (DA33135). Primer sets targeting the *aac(3)-IId* gene—responsible for the HR phenotype to TOB and the *tet*(A) gene also present on the plasmid—were used to assess gene dosage changes (Table S2). Quantification was normalized to the chromosomal *llp* gene, which served as a single-copy internal reference (Table S2). A GCN increase above two in a Keio mutant compared to DA33135 was considered as an increased gene dosage of plasmid-located genes, and only clones without increased gene dosage of plasmid genes *aac(3)-IId* and *tet*(A) were selected for HR phenotypic analysis and stored at −80°C in 10% DMSO. The final set of Keio constructs used for further investigation is listed in Table S1. The MICs of TOB were determined for all confirmed constructs and their corresponding parental Keio strains (with the cat cassette removed) using E-test strips (bioMérieux), following the manufacturer’s instructions.

### Population analysis profiling (PAP) test and HR determination

To evaluate the HR phenotype, *E. coli* recombination gene deletion mutants constructed via conjugation harboring the target plasmid and without detectable increase in GCN were selected for phenotypic screening. Three independent colonies per mutant were grown overnight in 100 µL MHB in 96-well plates, diluted 1:1,000, and subcultured once under the same conditions. Approximately 10⁸ CFU from each culture were plated onto MH agar containing increasing concentrations of TOB (0.25 mg/L–128 mg/L). For concentrations ≤16 mg/L, 5 µL drops were spotted in duplicate; for higher concentrations (32 mg/L–128 mg/L), 100 µL was spread evenly using sterile beads. Total viable counts were determined by plating serial dilutions (10⁻⁴ to 10⁻⁷) onto antibiotic-free MHA. After 48 h of incubation, HR frequency was calculated as (CFU/mL on antibiotic plate)/ (CFU/mL on antibiotic-free plate). Frequencies were plotted against antibiotic concentrations using GraphPad Prism (GraphPad Software, San Diego, CA, USA). Isolates were defined as HR when resistant subpopulations (≥10⁻⁷ frequency) grew at ≥8-fold higher TOB concentrations than those inhibiting the main population (1). The highest non-inhibitory concentration was defined as the greatest antibiotic concentration at which bacterial viability was reduced by no more than 80% relative to the total colony-forming units (CFU) on MHA plates without antibiotics.

### Isolation of resistant clones from subpopulations with HR phenotype

Antibiotic-resistant clones of all recombination gene deletion mutants carrying the plasmid, as well as the Keio wild-type strain with the plasmid, were selected from PAP test plates. Selection was performed on MH agar plates supplemented with TOB at two defined concentrations: (i) eightfold above the MIC of the main population, and (ii) the highest antibiotic concentration at which visible growth was observed for each isolate (maximum tested concentration was 128 mg/L TOB). The PAP tests were performed in triplicate, and for each experiment, two independent resistant clones per isolate were selected, resulting in a total of six independent clones per isolate and antibiotic concentration. Selected clones were re-isolated on TOB-containing plates and subsequently cultured overnight in MHB supplemented with the same antibiotic concentration as used during initial selection. Following overnight growth, 2 mL of culture was centrifuged, and the resulting bacterial pellet was stored at −20°C for genomic DNA extraction and further analysis. In parallel, an aliquot of each culture was stored in the in-house strain collection at −80°C. Detailed information regarding the specific antibiotic concentrations used for mutant selection is provided in Table S3.

### Quantification of GCN by ddPCR

The GCN associated with the HR phenotype toward TOB was quantified using the ddPCR method. Genomic DNA was extracted using a bead-beating protocol optimized for bacterial lysis, with minor modifications (43). Briefly, 340 μL of overnight culture was centrifuged, and the pellet resuspended in 250 μL of a 1:1 mixture of fast lysis buffer (Qiagen) and nuclease-free water. The suspension was transferred to cryotubes containing 0.75 g acid-washed glass beads (212–300 μm, Sigma-Aldrich) and 250 μL of phenol:chloroform:isoamyl alcohol (25:24:1). After 5 min on ice, samples were bead-beaten twice at 6.5 m/s for 20 s (with 2 min on ice between runs) using a FastPrep bead beater (MP Biomedicals). Lysates were centrifuged (8,000 rpm, 3 min, 4°C), and the aqueous phase was transferred to fresh tubes. A second extraction was performed with an equal volume of phenol:chloroform:isoamyl alcohol, followed by vortexing and centrifugation (13,000 rpm, 4 min, 4°C). The final aqueous phase containing purified DNA was used for ddPCR. The ddPCR was performed using the Bio-Rad QX200 system with EVAGreen chemistry. Each 24 μL reaction contained 12 μL of 2× EVAGreen master mix (Bio-Rad), 0.2 μL HindIII-HF (20,000 U/mL, NEB), 3 μL DNA template, 0.5 μL of each primer (100 nM final concentration), and 7.8 μL nuclease-free water. Reactions were incubated at room temperature for 10 min to allow digestion, followed by droplet generation using the Bio-Rad Automated Droplet Generator. PCR amplification was carried out under the following conditions: 95°C for 5 min; 40 cycles of 94°C for 30 s and 60°C for 1 min; followed by stabilization at 4°C for 10 min and 90°C for 5 min. Droplets were analyzed using the QX200 Droplet Reader, and data were processed with QuantaSoft Analysis Pro (v.1.0.569). For PCN determination, primers targeting the plasmid located *repB* gene were used. Resistance gene dosage was assessed using primers for *aac(3)-IId* gene normalized to the chromosomal *llp* gene as a single-copy reference. All primer sequences are listed in Table S2.

### Whole-genome sequencing (WGS) and sequence analysis

WGS was conducted on selected resistant clones to detect ddPCR-undetected mechanisms and verify the absence of other mutations. Genomic DNA was extracted from 1 mL overnight culture pelleted cells collected during mutant selection on PAP test plates using the MasterPure Complete DNA and RNA Purification Kit (Epicentre), following the manufacturer’s instructions. DNA concentration was quantified using a Qubit 2.0 fluorometer (Invitrogen). Paired-end WGS (≤800 bp paired-end library) was conducted by BGI (Warsaw, Poland) using the DNBseq platform. Short-read sequences were mapped to the parental Keio reference genome (BW25113, genome reference CP009273.1) obtained from the NCBI database for all recombination gene deletion strains. Mapping and variant calling, including identification of single-nucleotide polymorphisms, insertions/deletions, and structural variants, were performed using the CLC Genomics Workbench software (version 25.0.1, Qiagen). GCN changes resulting from tandem gene amplification, PCN variation, or transposition events were analyzed by calculating the ratio of sequencing coverage (depth) between the plasmid and the chromosome, as well as the coverage of putative amplified regions relative to their surrounding genomic regions.

## RESULTS

### Plasmid transfer in recombination gene-deficient mutants

Tandem amplification of the *aac(3)-IId* gene due to unequal crossing over between IS6 family transposase repeated sequences present on each side of the gene and located on plasmid pDA33135-139 was identified previously as the principal mechanism underlying TOB HR in the parental strain DA33135 (11). Thus, we chose this plasmid to investigate the impact of proteins involved in recombination on HR mediated by tandem amplifications. Plasmid pDA33135-139 was conjugated into 19 Keio knockout strains, each lacking different genes involved in recombination pathways (40), and in the Keio wild-type strain (Table S1). For unknown reasons, a *recF* deletion mutant failed to receive the plasmid from the intermediate conjugant *E. coli* MG1655 despite repeated attempts and was therefore excluded from our study. To confirm that the conjugation and selection process in the presence of tetracycline did not result in increased gene dosage, GCN of plasmid genes was determined by ddPCR (Table S4). Absolute copy numbers of the *aac(3)-IId* and *tet*(A) genes were quantified and normalized to the chromosomal single-copy *llp* gene. No gene dosage increase (>2-fold) was detected in any of the recombination gene deletion strains. The relative *aac(3)-IId* and *llp* ratio ranged from 0.80 to 1.57, and the *tet*(A) to *llp* ratio ranged from 0.74 to 1.35 among recombination gene deletion strains, both comparable to the parental isolate DA33135 (0.94–1.09 and 0.92–1.09, respectively), confirming that plasmid acquisition did not lead to increased gene dosage. All 19 recombination gene deletion constructs containing the plasmid along with the Keio wild-type strain were considered genetically stable and were used in subsequent experiments.

### Keio mutants carrying plasmid pDA33135-139 exhibit different levels of resistance to TOB

We assessed the impact of plasmid pDA33135-139 carrying the *aac(3)-IId* in the different strains on TOB MIC and observed variations in resistance levels. The highest MIC (6 mg/L) was observed in strains with deletions in *recR*, *recT*, *ruvABC*, and *sbcBCD*, while the lowest MIC (1.5 mg/L) was detected in *recA* and *recB* mutants, a value comparable to the Keio wild type carrying the plasmid (1 mg/L) (Fig. S1). Overall, most recombination gene deletion constructs showed a modest increase in resistance, with up to a 4-fold increase in MIC relative to the wild-type strain with the plasmid. When comparing each strain carrying the plasmid to the corresponding strain devoid of the plasmid, the MIC increase was more pronounced, ranging from 3-fold increase (*sbcB* mutant) to 12- to 16-fold increase in MIC (*recA*, *recB*, *recR*, and *sbcB* mutants) when the plasmid was present. The Keio wild-type, along with *recJ*, *recN*, and *recD* mutants, showed a consistent 4-fold increase in MIC upon acquisition of the plasmid (Fig. S1). Overall, these findings show that the recombination gene deletions can have effects on the basal level of resistance, independent of alterations in resistance GCN.

### HR phenotype persists across recombination gene knockouts

**Fig 2:**
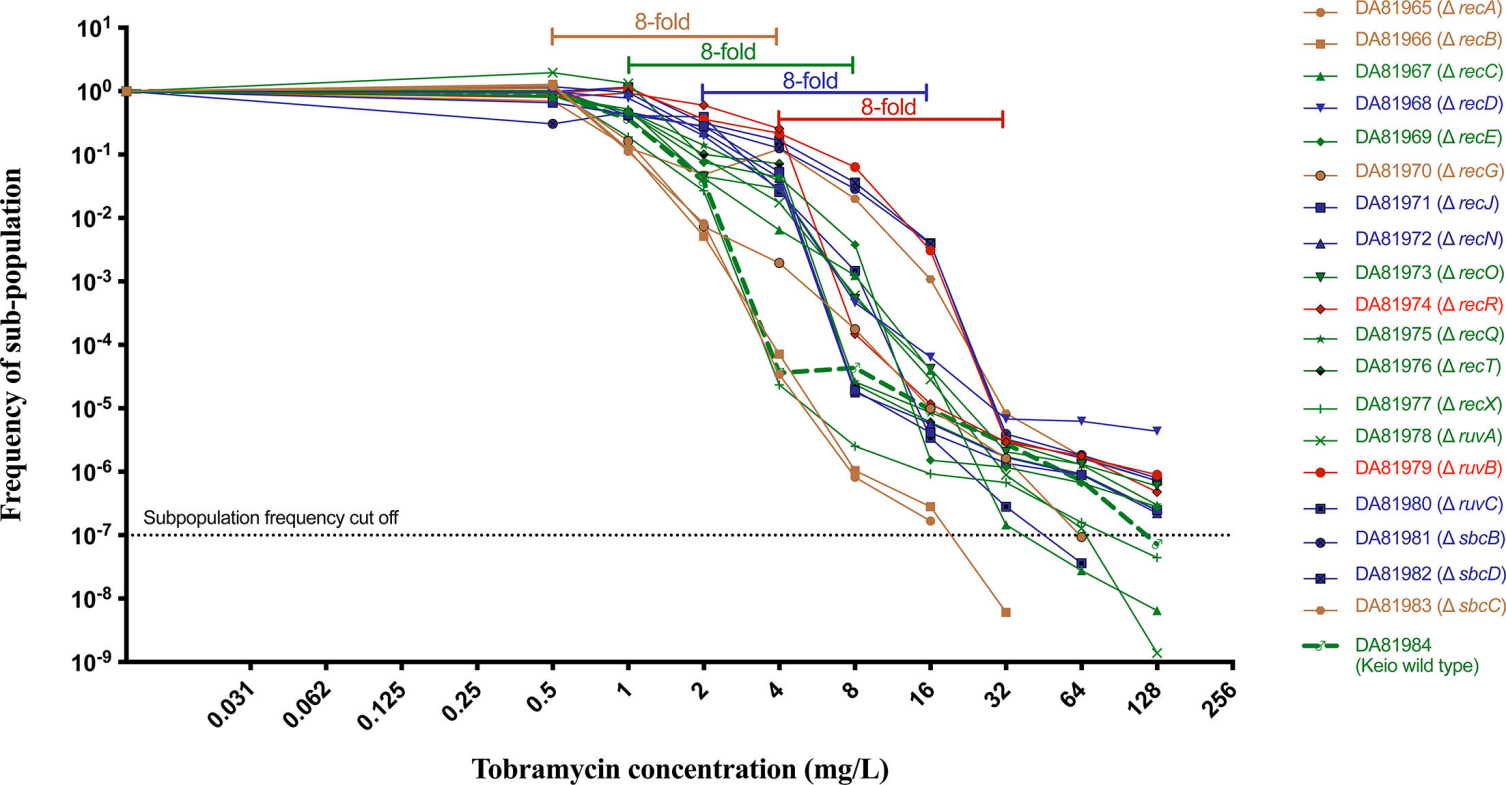
PAP curves in the presence of TOB for the Keio wild-type strain and Keio strains with deletions in recombination genes, all carrying plasmid pDA33135-139. Strains exhibiting the same HR thresholds of an eightfold increase in survival for the subpopulation compared to the main population not affected by TOB at a frequency ≥10⁻⁷ are color-coded in both the legend and scatter dot plot.

To assess whether recombination gene-deficient Keio strains carrying the plasmid exhibit HR to TOB, we performed PAP tests on all strains transformed with the plasmid. The HR phenotype was observed in the Keio wild-type strain and in all recombination gene deletion strains carrying the plasmid, indicating that disruption of the individual recombination genes did not prevent HR. Sixty percent (12/20) of the constructs showed growth of a subpopulation with a frequency equal to or above 10^−7^ at the highest antibiotic concentration tested (128 mg/L) in the PAP analysis (Fig. 2; Fig. S2). Interestingly, for *recA* and *recB* mutants, the subpopulations of mutants growing at frequency above 10^−7^ could not grow on TOB concentrations as high as for all other recombination gene deficient mutants (Fig. 2; Fig. S2). A majority of the recombination gene deletion strains, except strains with *recA* and *recB* deletions, exhibited increased subpopulation frequencies at different concentrations of TOB compared to the Keio wild-type strain (Fig. 2; Fig. S2). This is likely due to the lower MIC of the Keio wild-type isolate with the plasmid relative to most of the recombination gene deletion mutants (Fig. S1), resulting in a rightward shift of the PAP curve and less killing at higher antibiotic concentrations. These findings suggest that deletion of recombination genes other than *recA* and *recB* had no or little impact on HR and may even enhance resistant subpopulation frequencies at higher TOB concentrations.

Similarly to the results of the MIC tests, PAP tests showed that susceptibility to TOB varied across the different recombination gene deletion strains. In strains with deletions in *recR* and *ruv,* resistance of the main population reached 4 mg/L, which was higher than for the majority of strains (14 out of 20), for which the main populations had resistance level of 1 or 2 mg/L. In contrast, mutants in *recA*, *recB*, *recG*, and *sbcC* were more susceptible, as their main populations reached a resistance level of 0.5 mg/L only (Fig. 2; Fig. S2).

### Recombination gene deletions reduce tandem amplification-mediated HR

We investigated whether tandem amplification of the *aac(3)-IId* gene present on plasmid pDA33135-139 remains the primary HR mechanism in subpopulations of recombination gene-deficient strains exposed to high TOB concentrations. Using the different recombination gene-deficient strains, a total of 228 TOB-resistant mutants were selected and analyzed at eightfold above the highest TOB concentration that did not affect the main population (116 mutants) or at the highest TOB concentration allowing subpopulation growth at a frequency ≥10⁻⁷ (112 mutants) (Table S3).

**Fig 3:**
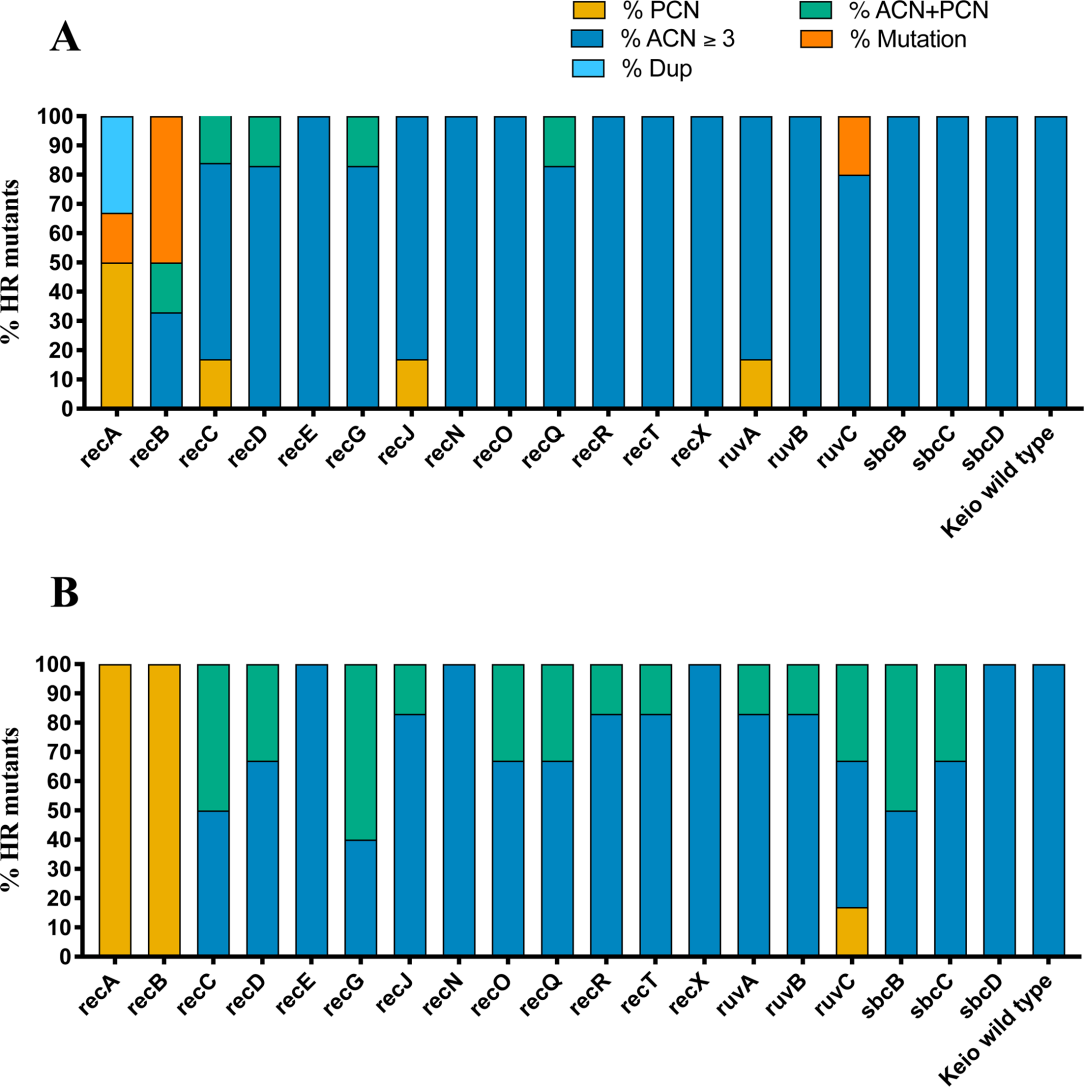
Genetic mechanisms underlying HR in Keio recombination gene deletion mutants carrying plasmid pDA33135-139. (**A**) TOB-resistant mutants selected at eightfold above the MIC of the main population, and (**B**) TOB-resistant mutants selected at the highest concentration permitting visible growth. For each gene deletion background, six independent mutants were analyzed except for *recN*, *recO*, *recR*, and *ruvC* in panel (**A**), where five mutants were analyzed, and for *recA*, *recB*, and *recG* in panel (**B**), where two, three, and five mutants were analyzed, respectively. For rec (resistance mechanisms were categorized as PCN increase, gene amplification (ACN), both (ACN + PCN), duplication (Dup), or mutation.

Analysis of the 116 mutants selected at eightfold by ddPCR revealed that 84.5% (98/116) exhibited tandem amplification of *aac(3)-IId* (copy number threshold >3), 5.2% (6/116) had increased PCN, and 4.3% (5/116) exhibited both tandem amplifications and increased PCN. Four mutants had no detectable increase in *aac(3)-IId* GCN. Mutants harboring deletions in recombination genes including *recE*, *recN*, *recO*, *recR*, *recT*, *recX*, *ruvB*, *sbcB*, *sbcC*, and *sbcD*, as well as the Keio wild-type strain carrying plasmid pDA33135-139, consistently demonstrated tandem amplification of *aac(3)-IId* as the sole resistance mechanism, without any detectable increase in PCN. In contrast, four out of six TOB-resistant mutants selected with the *recA* strain had no tandem gene amplifications, while two mutants had a large duplication carrying *aac(3)-IId* flanked by a single IS26 element and no repeated region at the opposite side of the amplified unit, as determined by WGS. In these mutants, resistance was primarily attributed to elevated PCN (Fig. 3A; Fig. S3).

Among the 112 mutants selected at the highest TOB concentrations, 73.2% (82/112) exhibited *aac(3)-IId* amplification alone, 5.4% (6/112) showed increased PCN alone, and 21.4% (24/112) had both mechanisms of increased gene dosage. Only one mutant lacked any increase in *aac(3)-IId* gene dosage. Notably, strains deficient in *recE*, *recN*, *recX, sbcD,* and the Keio wild-type had solely *aac(3)-IId* amplification as TOB resistance mechanism, indicating that resistance in these genetic backgrounds is likely mediated solely through tandem gene amplification (Fig. 3B; Fig. S3). It is also important to note that only two and three mutants were obtained from the *recA* and *recB* backgrounds, respectively (from triplicate PAP tests) at 16 mg/L whereas six or more mutants were recovered for the other constructs (Fig. 2B) at 32 mg/L or above. Comparative analysis of mutants selected under both TOB conditions revealed that the extent of tandem amplification varied across different mutants, ranging from 3- to 39-fold at 8-fold of TOB MIC to 5- to 83-fold at the highest concentration permitting growth. Furthermore, mutants with deletions of *recA*, *recB*, *recC*, *recj*, *ruvA*, and *ruvC* had noticeably disrupted or abolished tandem amplification of *aac(3)-IId*. In contrast, mutants lacking *recE*, *recN*, *recX*, *sbcD*, or the Keio wild-type strain consistently retained *aac(3)-IId* tandem amplification as the sole resistance mechanism, indicating that these genes are dispensable for the process. Collectively, these findings indicate that tandem gene amplification relies on RecA activity and on RecB and RecC activity to a lower extent. Other recombination gene deletions exerted minimal impact on tandem amplification dynamics (Fig. 3A and B; Fig. S3).

### Resistance profiles and gene dosage dynamics of HR-derived mutants

**Fig 4:**
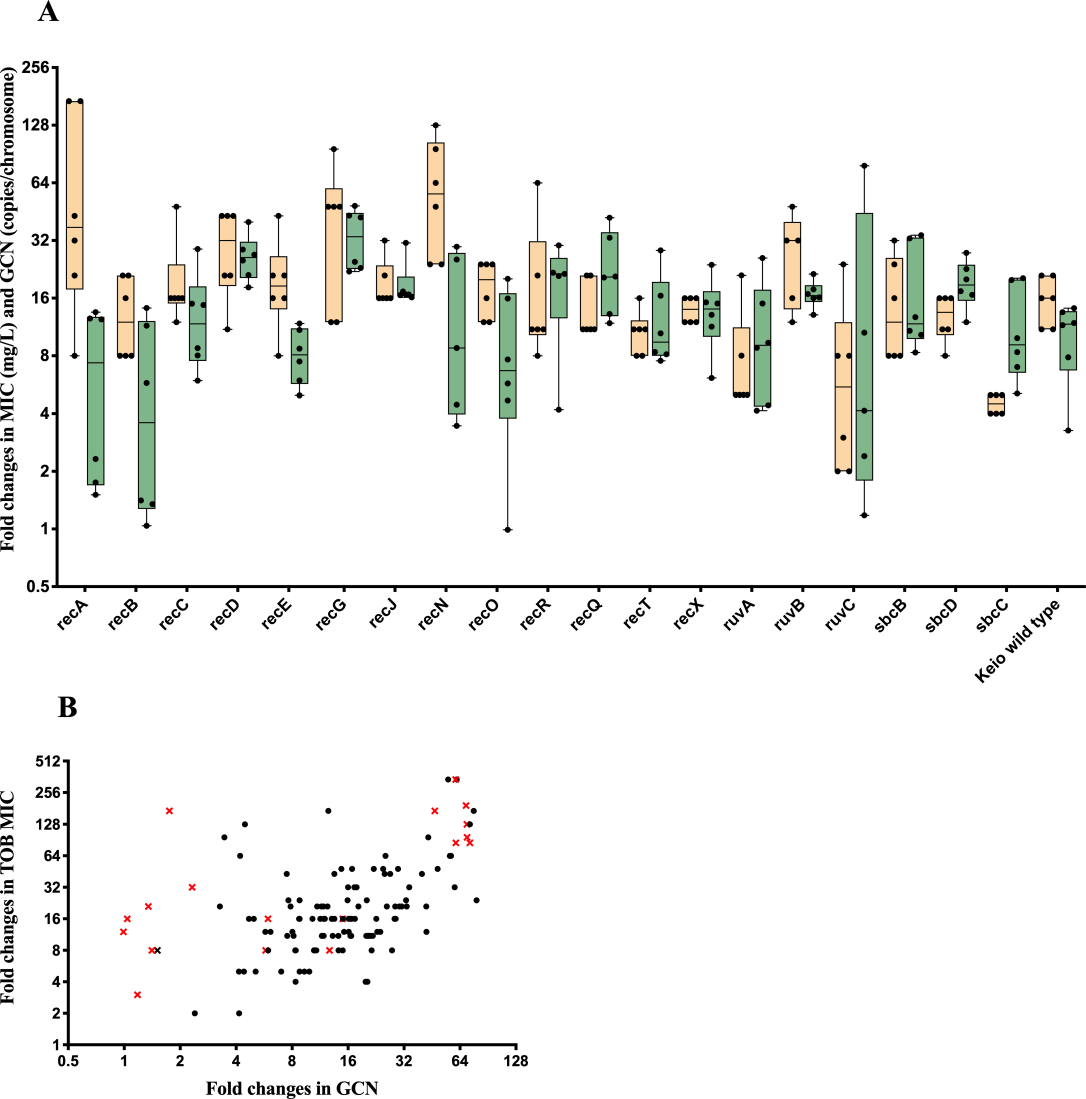
Fold changes in TOB MIC and GCN in mutants with deleted recombination genes. (**A**) Box plots showing fold changes in MIC of TOB (mg/L, yellow bars) and GCN (copies/chromosome, green bars) for resistant mutants isolated from recombination mutants. Mutants were selected at eightfold or above the MIC of the main population. Individual data points within each bar indicate individual resistant mutants isolated from the corresponding genetic background. The line inside each box represents the median, the box edges indicate the 25th and 75th percentiles, and the whiskers extend to the minimum and maximum values. (**B**) Correlation between fold changes in MIC and GCN across all mutants isolated at eightfold or above the TOB concentration of the main population. Mutants with chromosomal mutations were highlighted with a red cross in the figure. The correlation was assessed using Spearman’s rank test (*P* < 0.0001).

We investigated the *aac(3)-IId* gene dosage increase and level of TOB resistance in HR mutants, as determined by E-tests, compared to their respective parental Keio recombination gene deletion strains. A total of 120 resistant mutants isolated from PAP tests at 8-fold above the TOB concentration were analyzed. They exhibited significantly increased resistance, with MICs at least 2-fold above that of their respective parental strain. The highest MIC increases were observed in mutants isolated from strains with *recA, recG, and recN* deletions, with an average MIC increase of 63- to 93-fold. The smallest MIC increase was observed in mutants isolated from strains with *ruvA*, *ruvC*, and *sbcC* deletions, with the average MIC increasing only 5- to 8-fold. Resistant mutants derived from the Keio wild-type background exhibited a 16-fold MIC increase, comparable to mutants isolated from strains with *recB, recQ, recR*, *recX,* and *sbcD* deletions (Fig. 4A; Fig. S4). ddPCR analysis of mutants selected at 8-fold above MIC revealed *aac(3)-IId* gene dosage increases, either through tandem amplification or increased PCN and ranging from 5- to 27-fold (Fig. 4A; Fig. S4). Mutants lacking *recD* and *recG* exhibited the highest average relative gene dosage increases (20- and 27-fold, respectively) compared to the parental strain DA33135, whereas mutants with deletions in *recA*, *recB*, *recO*, *recE*, *sbcC*, or *ruvA* showed the lowest increases (5- to 9-fold), irrespective of the underlying mechanism.

To investigate whether increased *aac(3)-IId* gene dosage correlated with elevated TOB resistance, 15 additional mutants selected at TOB concentrations exceeding eightfold above the MIC of the main population were randomly selected and added to the 120 isolates previously selected at eightfold above the TOB concentration that did not affect the main population. Based on the ddPCR results obtained for all 135 analyzed mutants, Spearman’s rank correlation revealed a significant association between *aac(3)-IId* GCN and TOB MIC (*P* < 0.0001; Fig. 4B). However, no strong linear relation was observed across the entire data set (*r* = 0.478). When stratified by genetic background, mutants lacking *recA*, *recB*, *recC*, or *sbcB* included outliers with disproportionately high MICs despite minimal or undetectable GCN increases, indicating the presence of other resistance mechanisms (Fig. S4). This could involve either transcriptional changes induced by the recombination mutations and/or bona fide resistance mutations.

### WGS reveals alternative resistance pathways driven by point mutations

Among the 228 mutants that were analyzed with ddPCR for the detection of increased GCN, 28 isolates were also whole genome sequenced to identify additional mutations that might contribute to the resistance, particularly in outliers with low GCN but high MIC, and to confirm that increased MIC was solely attributable to GCN increase. These included nine isolates with discrepancies between GCN and MIC, four isolates without detectable *aac(3)-IId* GCN increase, and 15 isolates selected at TOB concentrations above eightfold of the TOB concentration that did not affect the main population and displaying *aac(3)-IId* tandem amplifications. Of the four isolates with no detectable *aac(3)-IId* GCN increase, three had point mutations in different genes and no *aac(3)-IId* GCN increase, confirming the ddPCR results; one isolate obtained from the *recO* mutant was excluded from further analysis due to the low quality of the sequencing data. Among the three analyzed isolates, two isolated from the *recB* mutant had identical mutations in *ykgE* and *ptrA* (Table S5). These mutations have no previously described link to antibiotic resistance. A mutant isolated from an isolate lacking *ruvC* carried a mutation in *fusA*, a gene previously described to be involved in aminoglycoside resistance in *E. coli* (44). For isolates that appeared as outliers in Fig. 4B (i.e. they have an increased MIC despite low GCN), WGS provided additional insights. In mutants isolated from the strain with a *recA* deletion, increased MIC was caused by mutations in several genes previously associated with aminoglycoside resistance, including *fusA*, *rpsL* (45), *ubiH* (46), and *kdpD* (47) (Table S5). In one mutant isolated from the strain carrying the *recB* deletion, a four-nucleotide insertion in *ubiF* likely impaired its function and explained the elevated MIC (48). In another mutant, a fourfold tandem amplification of the *aac(3)-IId* gene was present along with additional mutations in *aceE* and *ykgE***,** neither of which is known to contribute to TOB resistance. Two mutants from the *recC* deletion background were also sequenced: one because of its high TOB MIC with only minimal GCN increase, and the other to investigate potential resistance mechanisms beyond GCN amplification. Both carried mutations in *hemA*, a gene involved in heme biosynthesis and previously linked to aminoglycoside resistance (49) (Table S5). Analysis of the 15 mutants included in the correlation study and selected at >8-fold MIC (Fig. 4B) revealed that in six mutants, increased MIC of TOB was solely attributable to increased *aac(3)-IId* gene dosage. The remaining nine mutants carried additional mutations: two mutants selected with the *recJ* deletion strain had identical mutations in *cyoC* and *ylbE;* while *cyoC* has been implicated in aminoglycoside resistance (50, 51), the role of *ylbE* remains unclear (Table S5). Three mutants selected with the *recN* deletion strain had identical nonsense mutations in *moaA*, a gene involved in molybdenum cofactor biosynthesis, but not implicated in antibiotic resistance. Two mutants selected with the *recD* deletion strain carried identical mutations in *bax*, a gene not associated with aminoglycoside resistance. One mutant selected with the *recA* deletion strain had mutations in *ubiH* and *kdpD*, both of which contribute to aminoglycoside resistance (48). Finally, the single mutant selected with the *recQ* deletion strain had three different mutations: in *hemC***,** *fdnG*, and an insertion of 14 nucleotides in *dinB* (Table S5). Although the direct contribution of *fdnG* and *dinB* to antibiotic resistance is not well established, mutations in *hem* genes have been clearly associated with aminoglycoside resistance (52). It is worth noting that the observed mutations in genes previously associated with aminoglycoside resistance (*fusA*, *rpsL*, *ubiH*, *kdpD*, *cyoC*, and *hem* genes) could potentially contribute to TOB resistance and stabilize the resistance phenotype. While these mutations may confer full resistance, the isolates could still display a HR phenotype if additional compensatory mutations are acquired that decrease resistance and increase fitness.

## DISCUSSION

In this study, we determined the role of recombination proteins and pathways in the tandem amplification-mediated mechanism of HR. Among the diverse mechanisms underlying HR, tandem amplification increases the copy number of resistance-associated genes, thereby enhancing the expression of proteins that exhibit only weak activity against the antibiotic when expressed from genes present at a single copy. This mechanism has been identified as a predominant driver of HR, particularly in gram-negative bacteria (11, 15). For example, the mechanism behind aminoglycoside HR in clinical *E. coli* and *Klebsiella pneumoniae* was dominated by tandem gene amplifications of aminoglycoside resistance genes (11, 18).

Tandem amplification typically results from unequal crossing over between homologous sequences flanking the amplified unit, a process dependent on homologous recombination. Consequently, the activity of recombination proteins may influence both the frequency and stability of these amplification events, thereby modulating HR phenotypes. Based on this, we hypothesized that disruption of key recombination pathways could suppress amplification-driven HR, as previously demonstrated for *recA*-deficient mutants (38, 39).

We showed that deletion of individual genes encoding proteins involved in homologous recombination pathways did not abolish the HR phenotype; all recombination gene deletion strains carrying pDA33135-139 retained the HR phenotype. Interestingly, some of the recombination mutants showed a general increase in resistance, which could potentially be due to transcriptional effects on resistance-related functions (e.g., efflux). However, we observed a notable reduction of up to 2-log in the resistant subpopulation frequency in strains lacking *recA* and *recB*, compared to both wild-type and other recombination gene deletion strains. This finding aligns with previous studies by Díaz-Díaz et al. (38) and Anderson et al. (39), which reported reductions in HR frequency upon *recA* inactivation in response to β-lactam, quinolone, and aminoglycoside antibiotics. Interestingly, while Díaz-Díaz et al. described a complete reversion to susceptibility upon *recA* deletion, our data indicate that *recA*-deficient strains still exhibit HR, albeit at significantly lower frequencies. Indeed, the different results obtained here and in the study by Díaz-Díaz et al. are likely due to the presence of other RecA-independent HR mechanisms in our experimental setup, such as HR via PCN increase and chromosomal mutations.

Our findings show that TOB HR in most of the *E. coli* recombination gene deletion strains still arises predominantly through tandem amplification of the plasmid-encoded *aac(3)-IId* gene. However, deletion of key recombination genes *recA*, *recB*, and *recC* significantly impaired tandem amplification, shifting the resistance mechanism toward increased PCN and, to a lesser extent, point mutations. This shift was most pronounced in *recA* and *recB* mutants, which showed no tandem amplification even under elevated antibiotic pressure (Fig. 3B). Interestingly, two of the *recA*-deficient HR mutants had a duplication event bordered by an IS element on one side only, which did not involve RecA. Indeed, alternative duplication mechanisms involving single IS sequences, such as duplication by replicative transposition, have been described before (22). In addition, Morical et al. (53) also have described duplication formation via single-strand annealing, template switching, and nonhomologous end joining, particularly in genomic regions lacking extensive homologous repeats, which can also contribute to the type of rearrangements that we observed in these isolates. Interestingly, the lack of amplification beyond two copies in *recA*-deficient strains, a mechanism dependent on unequal crossing over between large duplicated amplified units, further emphasizes the central role of RecA not only for duplication formation but, in particular, for higher-level amplification. In *recB* and *recC* deletion mutants selected at eightfold above the TOB concentration that did not affect the main population (Fig. 3A), the frequency of HR by tandem amplification was reduced, and instead, PCN increase and point mutations became dominant HR mechanisms. This is consistent with previous reports showing that mutations in *recB* or *recC* result in a 100- to 1,000-fold reduction in conjugational (“sexual”) recombination and a strong sensitivity to DNA damage (54, 55). Because the RecBCD enzyme complex functions as the primary helicase/nuclease responsible for initiating most recombination events (25), the persistence of amplification in some *recB* and *recC* mutants suggests potential partial compensation by the RecFOR pathway. Indeed, this pathway, although primarily involved in the repair of single-stranded DNA gaps, becomes essential for nearly all non-DSB (double strand break) recombination events in the absence of RecBCD (24, 56). In bacteria that naturally lack RecBCD, such as *Thermus thermophilus* and the highly damage-resistant *Deinococcus radiodurans*, the RecFOR system is also capable of processing DSBs, often in cooperation with RecQ helicase and RecJ exonuclease (30, 57). Thus, the residual gene dosage increases observed in our *recB* and *recC* mutants may be attributed to recombination events mediated through the alternative RecFOR pathway.

Mutants lacking *recJ*, *ruvA*, and *ruvC* exhibited partial impairment of gene amplification. In these genetic backgrounds, the majority of resistant mutants still relied on tandem gene amplification as the primary HR mechanism, with approximately 20% (2 out of 12 analyzed mutants for each background) displaying alternative mechanisms. However, current evidence is insufficient to precisely determine how mutations in these genes influence the overall frequency of homologous recombination. Given their established roles in branch migration and Holliday junction resolution (25, 26, 58)**,** the final steps of the recombination process, disruption of these functions could compromise DNA integrity and thereby affect tandem amplification. In contrast, deletions in *recE*, *recN*, *recX*, and *sbcD* showed minimal impact, indicating that these proteins are dispensable for tandem amplification-driven HR. These differential effects underscore the complexity of recombination pathways and their context-dependent roles in resistance evolution.

In this study, we demonstrated that all recombination gene deletion strains analyzed maintained a HR phenotype, with subpopulations displaying increased but variable levels of resistance to aminoglycoside antibiotics, as revealed by PAP tests and MIC analysis. This was explained by the presence, in our system, of several TOB HR mechanisms with different dependencies in regard to recombination functions. We found that in the wild-type parental strain, tandem amplification was the only mechanism observed, whereas in mutants deleted for recombination genes, several additional HR mechanisms were seen, with different mechanisms dominating depending on the recombination gene deleted. For example, in the *recA* and *recB* mutants, tandem amplifications were prevented, and the increase in *aac(3)-IId* GCN was now mainly due to PCN increase. WGS of resistant mutants also revealed that point mutations contributed to the aminoglycoside HR phenotype, as previously described (10, 11). Several mutants, particularly those with low GCN but high MIC, carried mutations in *fusA*, known to confer resistance to fusidic acid (59) and gentamicin in *Staphylococcus aureus* (10) and *E. coli* (44). Mutations were also identified in the *rpsL* gene, frequently implicated in aminoglycoside resistance across diverse bacterial species (including ESKAPE pathogens) (45). Additional mutations in *ubiH* and *ubiF*, key genes in the ubiquinone biosynthesis pathway that functions as an electron and proton shuttle in the bacterial respiratory chain, were also identified (46). Since aminoglycoside uptake depends on the proton motive force, disruptions in electron transport can reduce antibiotic entry (49). Notably, *ubiF* mutations impair energy production and have been linked to increased antibiotic tolerance and persistence in *E. coli* (48). Mutations in *hemA* and *hemC* were also identified in the *recQ* and *recC* backgrounds, similar to previous findings where *hemH* (60) and *hemC* (10) mutations impaired electron transport and conferred aminoglycoside resistance. Mutations in *cyoC*, which encodes a cytochrome oxidase subunit involved in cellular respiration, have previously been associated with aminoglycoside resistance in *E. coli* when downregulated. In addition, we observed mutations in *kdpD*, a gene frequently altered under both macrolide and aminoglycoside stress (50, 51), which may contribute to aminoglycoside resistance. The roles of *fdnG* and *ylbE* mutations in antibiotic resistance, however, remain unclear. Insertion in *dinB*, which encodes DNA polymerase IV and functions in the SOS response by bypassing DNA damage during replication, may indirectly contribute to resistance. These results show that, alongside tandem gene amplification, other pathways that are independent of the mutated recombination functions can contribute to HR toward aminoglycosides in *E. coli*. While resistance by point mutations in chromosomal genes is likely to be possible in most, if not all, *E. coli* isolates and other pathogens, PCN increase is dependent on the presence of a resistance gene on a plasmid that can increase in copy number under antibiotic selection pressure.

Correlation analysis between GCN and MIC among resistant HR mutants isolated at ≥8-fold above the highest TOB concentration not affecting the main population in the PAP assay showed a significant association. This relationship between GCN and MIC was consistent with previous observations in amikacin-resistant mutants selected with aminoglycosides, as well as in β-lactamase-mediated resistance to β-lactam antibiotics (17). However, the correlation was not linear across all recombination gene-deficient backgrounds for TOB. Previous reports have suggested that a linear correlation between β-lactamase copy number and MIC is often assumed for β-lactams (61), yet Nicoloff et al. demonstrated that such linearity does not consistently hold for certain β-lactams, including ceftazidime–avibactam and ertapenem, and aminoglycosides such as amikacin (17). Consistent with this, our results reinforce that linearity may not consistently occur for aminoglycosides either. It is worth noting that our correlation analysis is based exclusively on ddPCR data, which provides a robust measure of GCN. However, additional genetic changes within individual isolates may also contribute to the observed outcomes and may have caused the non-linearity that we observed. As WGS was not performed for all resistant mutants, the influence of such additional mutations cannot be excluded. Nevertheless, the prevalence of such mutations was assessed in a subset of TOB-resistant mutants, which revealed that among 15 mutants showing *aac(3)-IId* tandem amplification by ddPCR, 60% (9/15) had additional mutations, some of which are known to confer aminoglycoside resistance.

### Conclusion

Previous studies have implicated RecA in the HR phenotype, and our work expands our knowledge regarding 19 recombination-associated proteins with respect to their impact on tandem-amplification-associated HR. Our results establish *recA*, *recB*, and *recC* as critical mediators of this HR mechanism. The identification of alternative resistance pathways through either increased PCN or mutations in core genes underscores the need for multifaceted strategies to combat HR. Thus, targeting the recombination machinery may offer a promising avenue to suppress amplification-driven resistance and HR, but our data show that HR could still emerge through other mechanisms.

## AFTER EPUB

*[This article was published on 12 January 2026 with errors in Fig. 4B and the associated text and legend. The errors were corrected in the current version, posted on 14 January 2026.]*

## SUPPLEMENTAL MATERIAL

Supplemental Material mbio.03674-25-s0001.pdf Figures S1 to S4; Tables S1 to S5.

ASM does not own the copyrights to Supplemental Material that may be linked to, or accessed through, an article. The authors have granted ASM a non-exclusive, world-wide license to publish the Supplemental Material files. Please contact the corresponding author directly for reuse.
