# Supplementary material for: Deletions of recombination genes impair tandem amplification and reshape heteroresistance mechanisms in Escherichia coli
Source: mBio. 2026 Jan 12;17(2):e03674-25. doi: 10.1128/mbio.03674-25 (PMC12892994; doi:10.1128/mbio.03674-25)
Supplement: Supplemental Material — Figures S1 to S4; Tables S1 to S5. [file mbio.03674-25-s0001.pdf]

Supplementary material Heidarian et al.

Supplementary figures

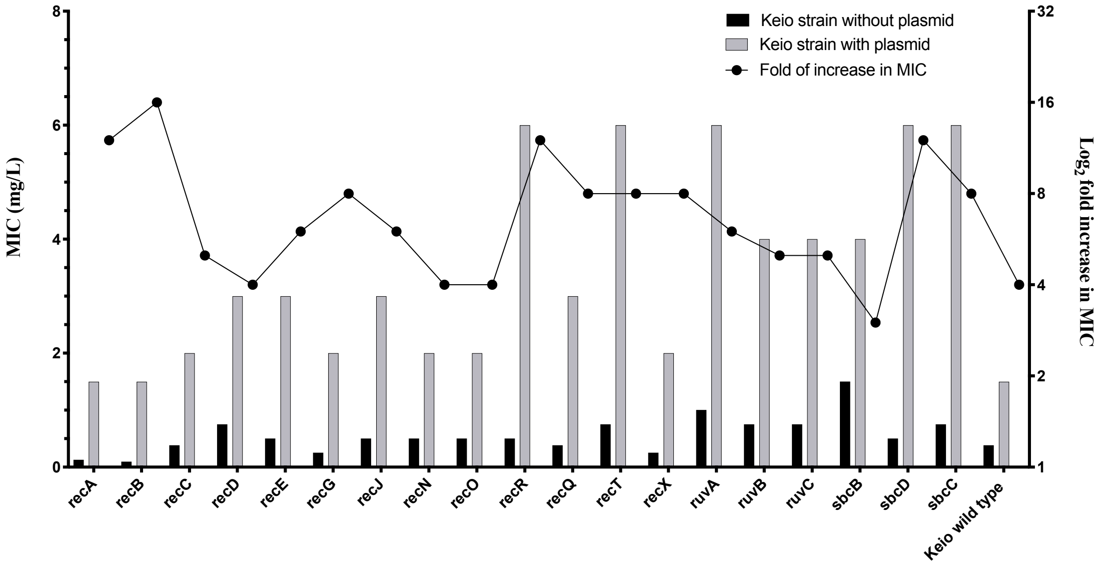

Supplementary Figure 1. Tobramycin MICs of final Keio constructs carrying plasmid p139 compared with their parental Keio strains without plasmid pDA33135-139. Bars show MIC values (left y-axis); dots indicate fold change relative to the parental strain (right y-axis).

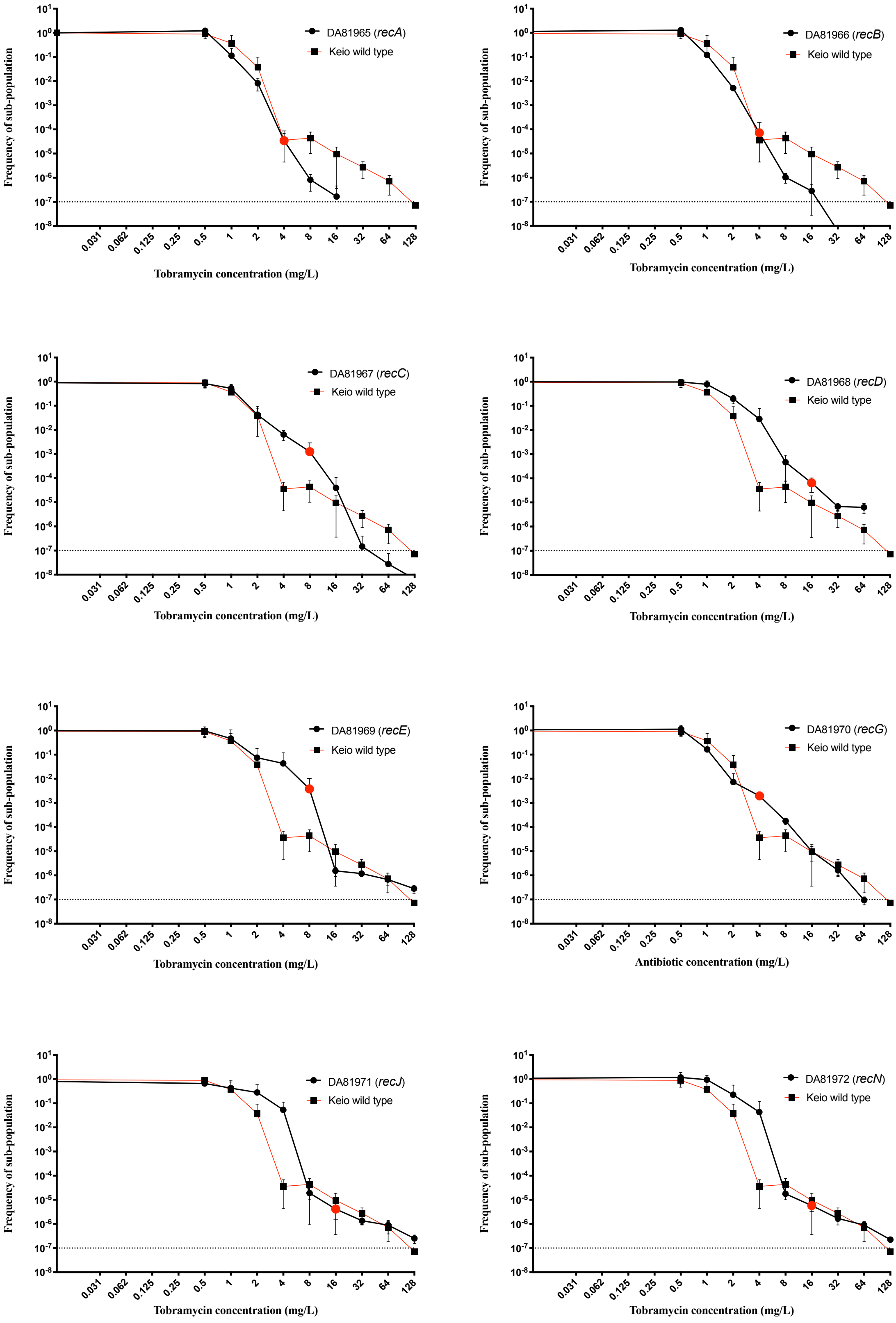
DA81965 (*recA*)
Keio wild type
DA81966 (*recB*)
Keio wild type
DA81967 (*recC*)
Keio wild type
DA81968 (*recD*)
Keio wild type
DA81969 (*recE*)
Keio wild type
DA81970 (*recG*)
Keio wild type
DA81971 (*recJ*)
Keio wild type
DA81972 (*recN*)
Keio wild type
Frequency of sub-population
Tobramycin concentration (mg/L)
Antibiotic concentration (mg/L)
0.031
0.062
0.125
0.25
0.5
1
2
4
8
16
32
64
128
10^{1}
10^{0}
10^{-1}
10^{-2}
10^{-3}
10^{-4}
10^{-5}
10^{-6}
10^{-7}
10^{-8}

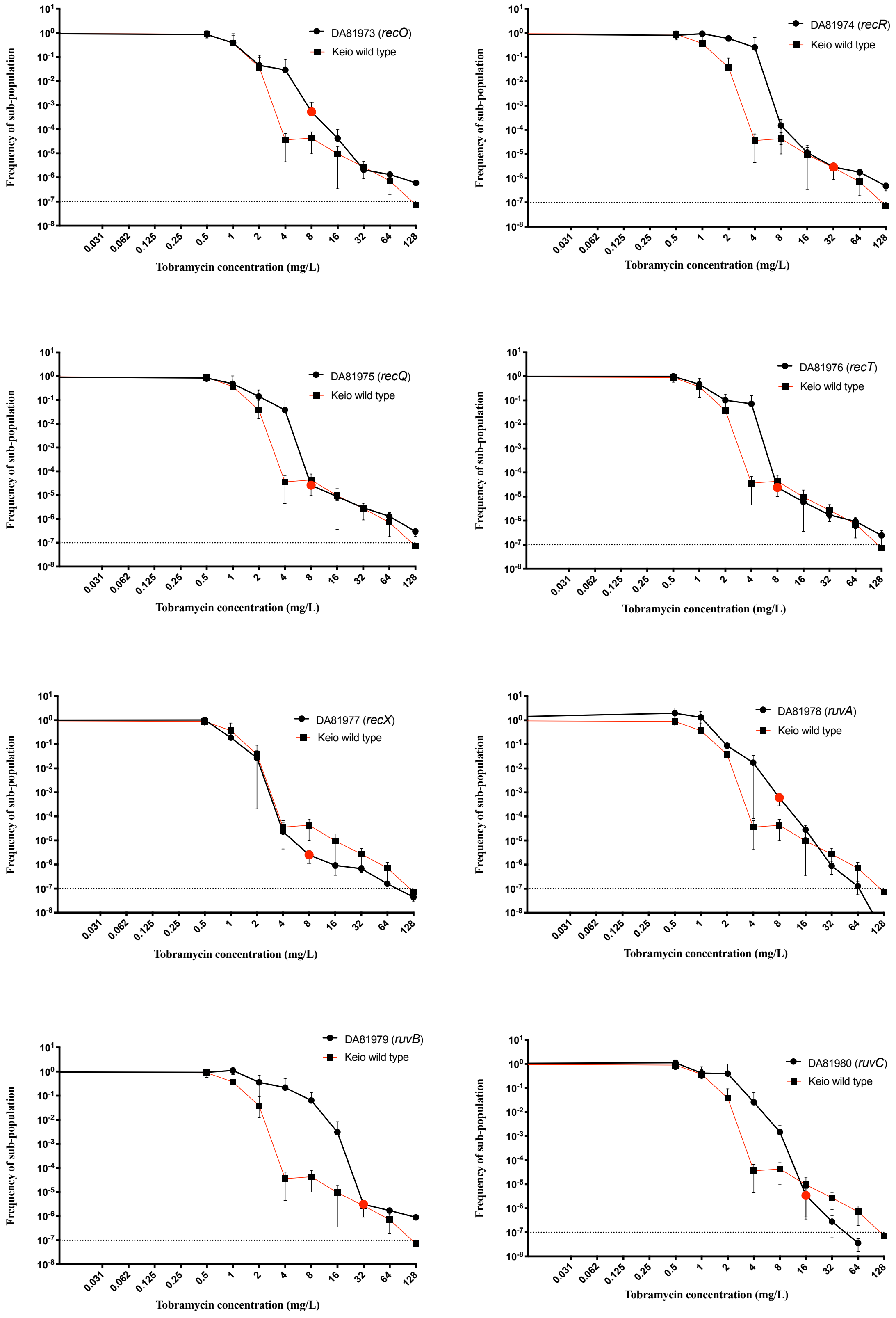
DA81973 (*recO*)
Keio wild type
Frequency of sub-population
Tobramycin concentration (mg/L)
DA81974 (*recR*)
Keio wild type
Frequency of sub-population
Tobramycin concentration (mg/L)
DA81975 (*recQ*)
Keio wild type
Frequency of sub-population
Tobramycin concentration (mg/L)
DA81976 (*recT*)
Keio wild type
Frequency of sub-population
Tobramycin concentration (mg/L)
DA81977 (*recX*)
Keio wild type
Frequency of sub-population
Tobramycin concentration (mg/L)
DA81978 (*ruvA*)
Keio wild type
Frequency of sub-population
Tobramycin concentration (mg/L)
DA81979 (*ruvB*)
Keio wild type
Frequency of sub-population
Tobramycin concentration (mg/L)
DA81980 (*ruvC*)
Keio wild type
Frequency of sub-population
Tobramycin concentration (mg/L)

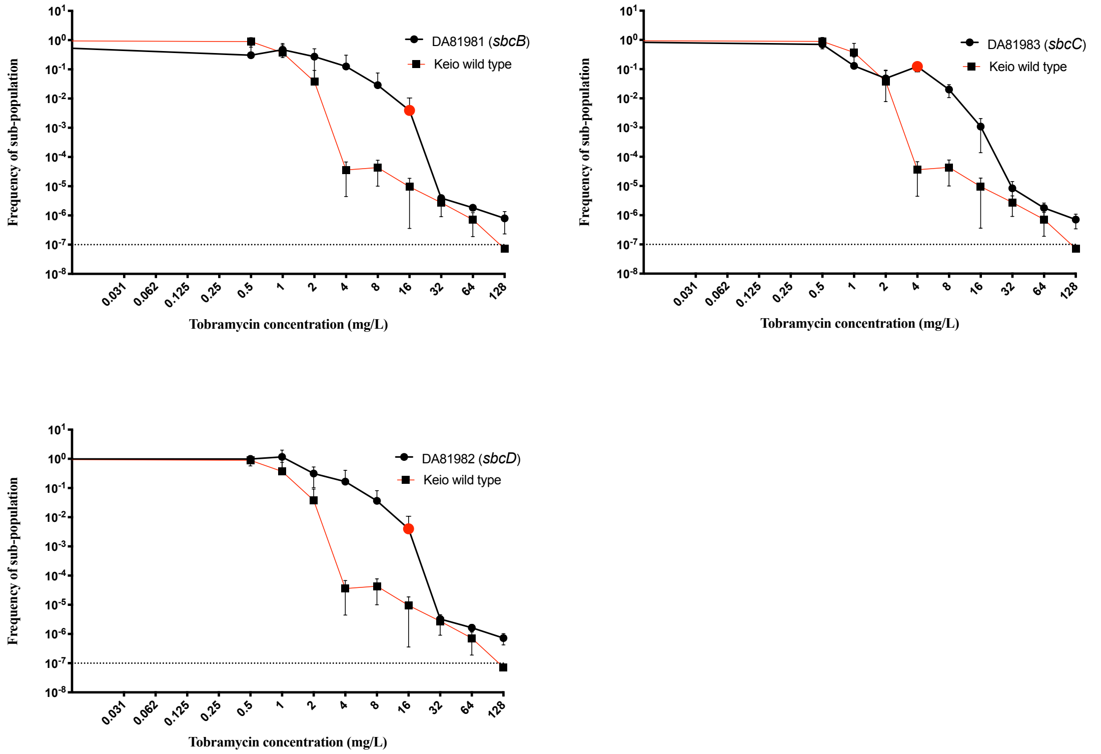

Supplementary Figure 2. Population analysis profiles (PAP) of Keio strains carrying plasmid pDA33135-139. Each curve represents the mean of three independent experiments. In each plot, the red curve corresponds to the Keio wild-type strain with plasmid pDA33135-139 (control) for comparison with their respective Keio recombination gene knockout strain. The tobramycin concentration corresponding to 8×MIC, which did not affect the main population but selected for the heteroresistant subpopulation, is highlighted in red on each curve. The horizontal dotted line marks the subpopulation frequency threshold (1×10^{-7}) used to define heteroresistant isolates. The standard deviation represents the variability among three independent biological replicates

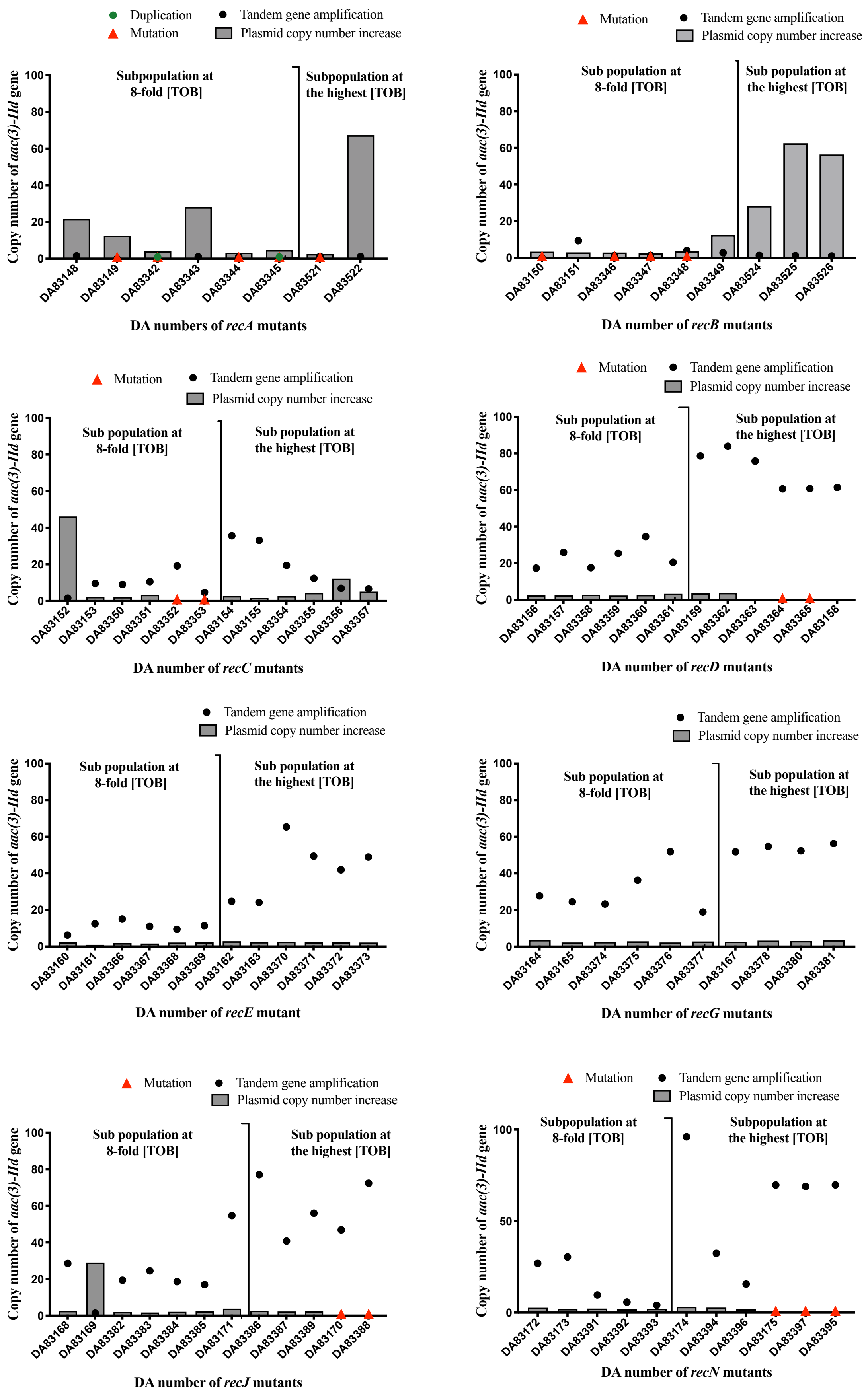
Duplication
Mutation
Tandem gene amplification
Plasmid copy number increase
Copy number of aac(3)-IId gene
Subpopulation at 8-fold [TOB]
Subpopulation at the highest [TOB]
DA83148 DA83149 DA83342 DA83343 DA83344 DA83345 DA83521 DA83522
DA numbers of recA mutants
Mutation
Tandem gene amplification
Plasmid copy number increase
Sub population at 8-fold [TOB]
Sub population at the highest [TOB]
DA83150 DA83151 DA83346 DA83347 DA83348 DA83349 DA83524 DA83525 DA83526
DA number of recB mutants
DA83152 DA83153 DA83350 DA83351 DA83352 DA83353 DA83154 DA83155 DA83354 DA83355 DA83356 DA83357
DA number of recC mutants
DA83156 DA83157 DA83358 DA83359 DA83360 DA83361 DA83159 DA83362 DA83363 DA83364 DA83365 DA83158
DA number of recD mutants
DA83160 DA83161 DA83366 DA83367 DA83368 DA83369 DA83162 DA83163 DA83370 DA83371 DA83372 DA83373
DA number of recE mutant
DA83164 DA83165 DA83374 DA83375 DA83376 DA83377 DA83167 DA83378 DA83380 DA83381
DA number of recG mutants
DA83168 DA83169 DA83382 DA83383 DA83384 DA83385 DA83171 DA83386 DA83387 DA83389 DA83170 DA83388
DA number of recJ mutants
DA83172 DA83173 DA83391 DA83392 DA83393 DA83174 DA83394 DA83396 DA83175 DA83397 DA83395
DA number of recN mutants

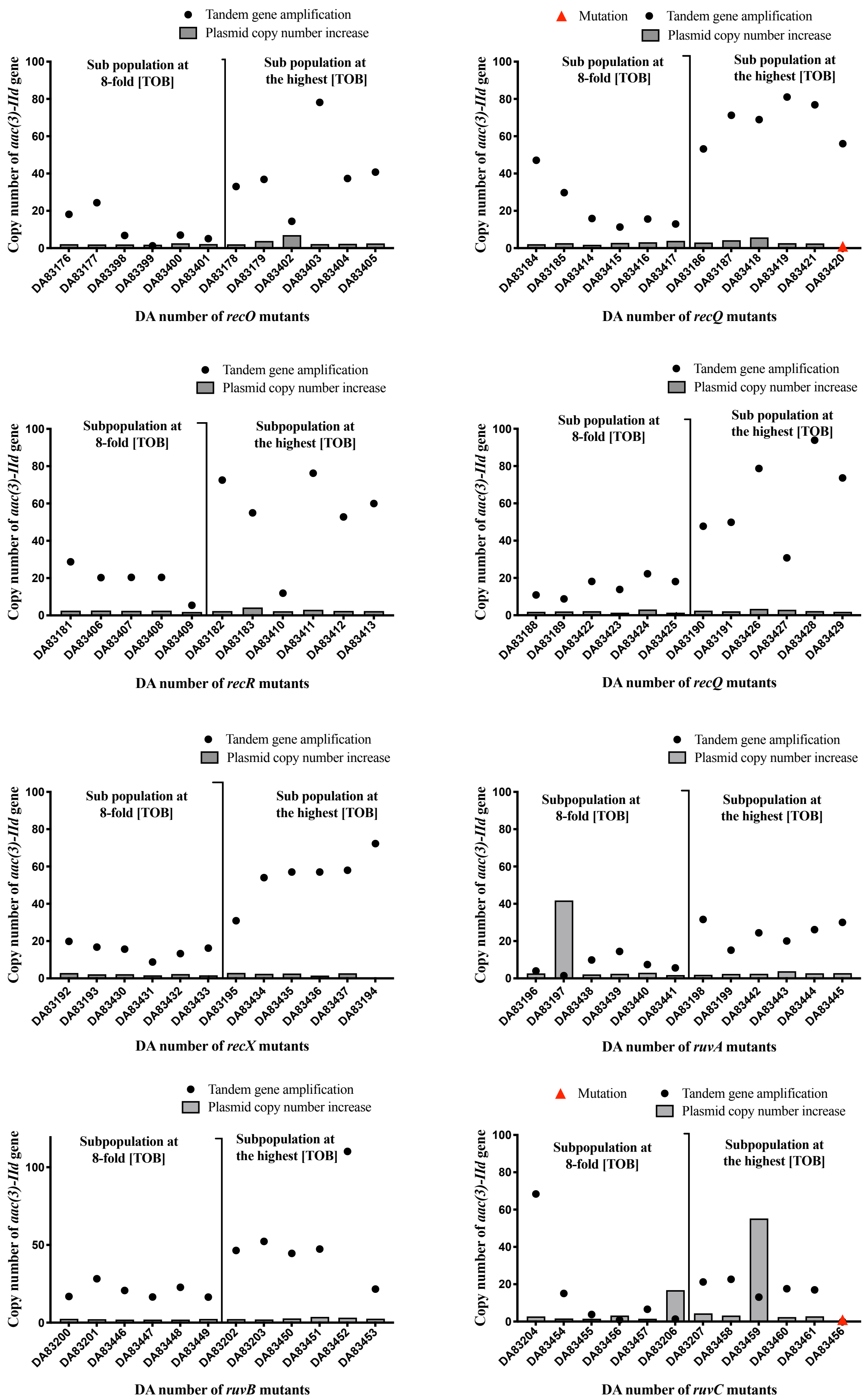
Tandem gene amplification
Plasmid copy number increase
Copy number of aac(3)-IId gene
Sub population at 8-fold [TOB]
Sub population at the highest [TOB]
DA83176 DA83177 DA83398 DA83399 DA83400 DA83401 DA83178 DA83179 DA83402 DA83403 DA83404 DA83405
DA number of recO mutants
Mutation
Tandem gene amplification
Plasmid copy number increase
Copy number of aac(3)-IId gene
Sub population at 8-fold [TOB]
Sub population at the highest [TOB]
DA83184 DA83185 DA83414 DA83415 DA83416 DA83417 DA83186 DA83187 DA83418 DA83419 DA83421 DA83420
DA number of recQ mutants
Tandem gene amplification
Plasmid copy number increase
Copy number of aac(3)-IId gene
Subpopulation at 8-fold [TOB]
Subpopulation at the highest [TOB]
DA83181 DA83406 DA83407 DA83408 DA83409 DA83182 DA83183 DA83410 DA83411 DA83412 DA83413
DA number of recR mutants
Tandem gene amplification
Plasmid copy number increase
Copy number of aac(3)-IId gene
Sub population at 8-fold [TOB]
Sub population at the highest [TOB]
DA83188 DA83189 DA83422 DA83423 DA83424 DA83425 DA83190 DA83191 DA83426 DA83427 DA83428 DA83429
DA number of recQ mutants
Tandem gene amplification
Plasmid copy number increase
Copy number of aac(3)-IId gene
Sub population at 8-fold [TOB]
Sub population at the highest [TOB]
DA83192 DA83193 DA83430 DA83431 DA83432 DA83433 DA83195 DA83434 DA83435 DA83436 DA83437 DA83194
DA number of recX mutants
Tandem gene amplification
Plasmid copy number increase
Copy number of aac(3)-IId gene
Subpopulation at 8-fold [TOB]
Subpopulation at the highest [TOB]
DA83196 DA83197 DA83438 DA83439 DA83440 DA83441 DA83198 DA83199 DA83442 DA83443 DA83444 DA83445
DA number of ruvA mutants
Tandem gene amplification
Plasmid copy number increase
Copy number of aac(3)-IId gene
Subpopulation at 8-fold [TOB]
Subpopulation at the highest [TOB]
DA83200 DA83201 DA83446 DA83447 DA83448 DA83449 DA83202 DA83203 DA83450 DA83451 DA83452 DA83453
DA number of ruvB mutants
Mutation
Tandem gene amplification
Plasmid copy number increase
Copy number of aac(3)-IId gene
Subpopulation at 8-fold [TOB]
Subpopulation at the highest [TOB]
DA83204 DA83454 DA83455 DA83456 DA83457 DA83206 DA83207 DA83458 DA83459 DA83460 DA83461 DA83456
DA number of ruvC mutants

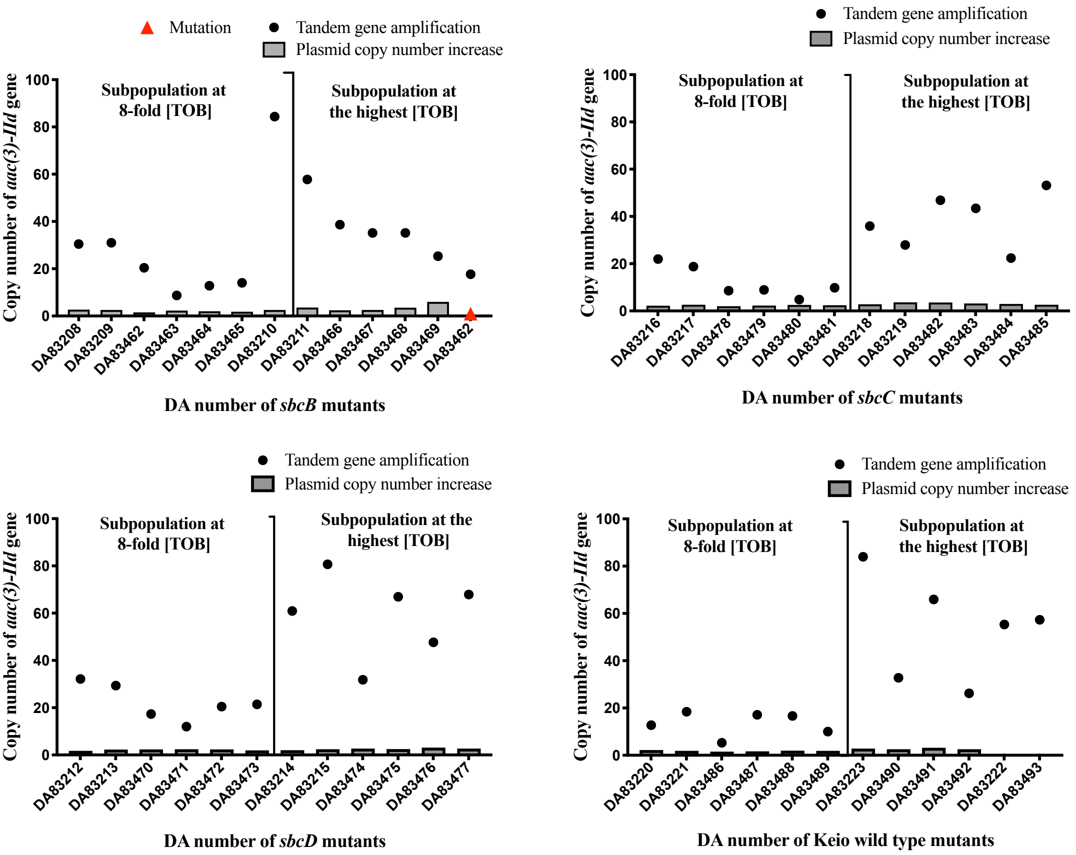

Supplementary Figure 3. Comparative analysis of resistance mechanisms in Keio mutants carrying plasmid pDA33135-139 under tobramycin selective pressure. Resistance mechanism behind gene copy number increase (tandem gene amplification and/or plasmid copy number increase) in Keio recombination gene deletion mutants harbouring plasmid pDA33135-139 selected at two TOB concentrations: 8-fold above the TOB concentration did not affect the growth of the main population and the highest tested concentration permitting visible growth of subpopulations at a frequency $\geq 10^{-7}$. Each black dot represents the ratio of the *aac(3)-IId* gene to the plasmid-located *repB* gene, indicating tandem gene amplification, while the green dot denotes a duplication observed only in a subset of *recA* mutants. Bars show the ratio of the *repB* gene (outside the amplification region on plasmid pDA33135-139) to the chromosomally encoded *llp* gene, reflecting plasmid copy number increase. Red triangles indicate mutations identified by whole-genome sequencing.

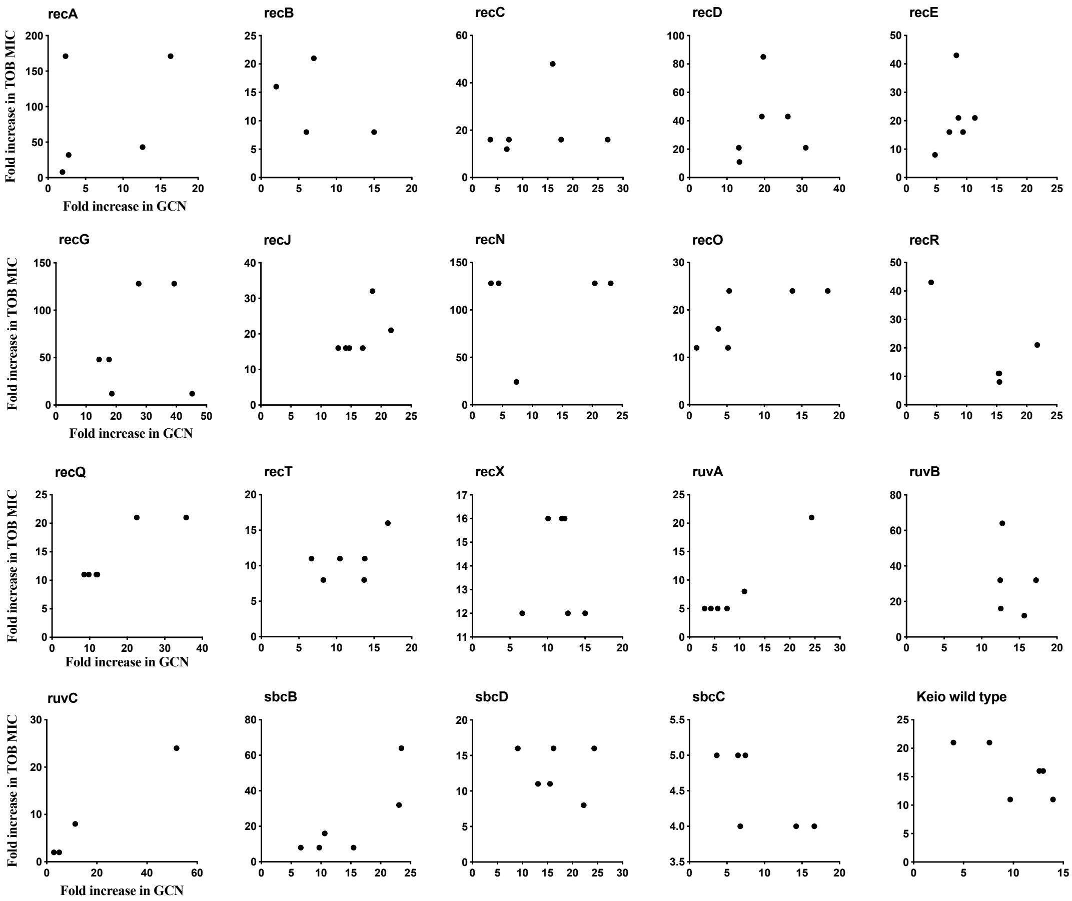

Supplementary Figure 4. Relationship between gene copy number (GCN) increase and tobramycin MIC in selected resistant mutants. Scatter plots show the fold increase in gene copy number (GCN) versus fold increase in resistance level (MIC) for resistant mutants selected from Keio strains with deletions in recombination-related genes at 8-fold above the MIC of the main population. Each panel represents data from one recombination deletion strain, and the Keio wild-type strain. Each dot refers to one resistant mutant.

Supplementary tables

Supplementary Table 1. List of all *E. coli* isolates used in this study. Twenty Keio strains with deletions in recombination genes, along with the parental wild-type strain BW25113 are listed. The identifier in parentheses corresponds to the designation from the original Keio collection. In the constructed Keio strains carrying plasmid p139, the kanamycin resistance cassette was excised leaving an FRT scar.

Strain ID (In-house collection)	Deleted gene	Genotype
***E. coli* Keio collection**		
K333 (JW2669)	*recA*	F-, *Δ(araD-araB)567*, *ΔlacZ4787*(::rrnB-3), λ^-, *ΔrecA774::kan*, *rph-1*, *Δ(rhaD-rhaB)568*, *hsdR514*
K3921(JW2788)	*recB*	F-, *Δ(araD-araB)567*, *ΔlacZ4787*(::rrnB-3), λ^-, *ΔrecB745::kan*, *rph-1*, *Δ(rhaD-rhaB)568*, *hsdR514*
K3933 (JW2790)	*recC*	F-, *Δ(araD-araB)567*, *ΔlacZ4787*(::rrnB-3), λ^-, *ΔrecC747::kan*, *rph-1*, *Δ(rhaD-rhaB)568*, *hsdR514*
K3630 (JW2787)	*recD*	F-, *Δ(araD-araB)567*, *ΔlacZ4787*(::rrnB-3), λ^-, *ΔrecD744::kan*, *rph-1*, *Δ(rhaD-rhaB)568*, *hsdR514*
K2185 (JW1344)	*recE*	F-, *Δ(araD-araB)567*, *ΔlacZ4787*(::rrnB-3), λ^-, *ΔrecE787::kan*, *rph-1*, *Δ(rhaD-rhaB)568*, *hsdR514*
K3416 (JW3677)	*recF*	F-, *Δ(araD-araB)567*, *ΔlacZ4787*(::rrnB-3), λ^-, *rph-1*, *ΔrecF735::kan*, *Δ(rhaD-rhaB)568*, *hsdR514*
K2710 (JW3627)	*recG*	F-, *Δ(araD-araB)567*, *ΔlacZ4787*(::rrnB-3), λ^-, *rph-1*, *ΔrecG756::kan*, *Δ(rhaD-rhaB)568*, *hsdR514*
K2212 (JW2860)	*recJ*	F-, *Δ(araD-araB)567*, *ΔlacZ4787*(::rrnB-3), λ^-, *ΔrecJ743::kan*, *rph-1*, *Δ(rhaD-rhaB)568*, *hsdR514*
K3405 (JW5416)	*recN*	F-, *Δ(araD-araB)567*, *ΔlacZ4787*(::rrnB-3), λ^-, *ΔrecN772::kan*, *rph-1*, *Δ(rhaD-rhaB)568*, *hsdR514*
K3403 (JW2549)	*recO*	F-, *Δ(araD-araB)567*, *ΔlacZ4787*(::rrnB-3), λ^-, *ΔrecO737::kan*, *rph-1*, *Δ(rhaD-rhaB)568*, *hsdR514*
K3780 (JW5855)	*recQ*	F-, *Δ(araD-araB)567*, *ΔlacZ4787*(::rrnB-3), λ^-, *rph-1*, *ΔrecQ767::kan*, *Δ(rhaD-rhaB)568*, *hsdR514*
K1560 (JW0461)	*recR*	F-, *Δ(araD-araB)567*, *ΔlacZ4787*(::rrnB-3), *Δ*RecR776::kan, λ-, rph-1, Δ(rhaD-rhaB)568, hsdR514
K1566 (JW1343)	*recT*	F-, *Δ(araD-araB)567*, *ΔlacZ4787*(::rrnB-3), λ^-, *ΔrecT786::kan*, *rph-1*, *Δ(rhaD-rhaB)568*, *hsdR514*
K3661 (JW2668)	*recX*	F-, *Δ(araD-araB)567*, *ΔlacZ4787*(::rrnB-3), λ^-, *ΔrecX773::kan*, *rph-1*, *Δ(rhaD-rhaB)568*, *hsdR514*
K1571 (JW1850)	*ruvA*	F-, *Δ(araD-araB)567*, *ΔlacZ4787*(::rrnB-3), λ^-, *ΔruvA786::kan*, *rph-1*, *Δ(rhaD-rhaB)568*, *hsdR514*
K1160 (JW1849)	*ruvB*	F-, *Δ(araD-araB)567*, *ΔlacZ4787*(::rrnB-3), λ^-, *ΔruvB785::kan*, *rph-1*, *Δ(rhaD-rhaB)568*, *hsdR514*
K1572 (JW1852)	*ruvC*	F-, *Δ(araD-araB)567*, *ΔlacZ4787*(::rrnB-3), λ^-, *ΔruvC789::kan*, *rph-1*, *Δ(rhaD-rhaB)568*, *hsdR514*
K2537 (JW1993)	*sbcB*	F-, *Δ(araD-araB)567*, *ΔlacZ4787*(::rrnB-3), λ^-, *ΔsbcB780::kan*, *rph-1*, *Δ(rhaD-rhaB)568*, *hsdR514*
K1558 (JW0387)	*sbcC*	F-, *Δ(araD-araB)567*, *ΔlacZ4787*(::rrnB-3), *ΔsbcC761::kan*, λ^-, *rph-1*, *Δ(rhaD-rhaB)568*, *hsdR514*
K2025 (JW0388)	*sbcD*	F-, *Δ(araD-araB)567*, *ΔlacZ4787*(::rrnB-3), *ΔsbcD762::kan*, λ^-, *rph-1*, *Δ(rhaD-rhaB)568*, *hsdR514*
DA28696 (BW25113)	Keio *wildtype*	F-, *Δ(araD-araB)567*, *ΔlacZ4787*(::rrnB-3), λ^-, *rph 1*, *Δ(rhaD-rhaB)568*, *hsdR514,* [p139]
**Constructed Keio strains carrying plasmid pDA33135-139**		
DA81965	*recA*	F-, *Δ(araD-araB)567*, *ΔlacZ4787*(::rrnB-3), λ^-, *ΔrecA774*, *rph-1*, *Δ(rhaD-rhaB)568*, *hsdR514,* [p139]

DA81966	*recB*	F-, *Δ(araD-araB)567*, *ΔlacZ4787*(::rrnB-3), λ^-, *ΔrecB745*, *rph-1*, *Δ(rhaD-rhaB)568*, *hsdR514,* [p139]
DA81967	*recC*	F-, *Δ(araD-araB)567*, *ΔlacZ4787*(::rrnB-3), λ^-, *ΔrecC747*, *rph-1*, *Δ(rhaD-rhaB)568*, *hsdR514,* [p139]
DA81968	*recD*	F-, *Δ(araD-araB)567*, *ΔlacZ4787*(::rrnB-3), λ^-, *ΔrecD744*, *rph-1*, *Δ(rhaD-rhaB)568*, *hsdR514,* [p139]
DA81969	*recE*	F-, *Δ(araD-araB)567*, *ΔlacZ4787*(::rrnB-3), λ^-, *ΔrecE787*, *rph-1*, *Δ(rhaD-rhaB)568*, *hsdR514,* [p139]
DA81970	*recG*	F-, *Δ(araD-araB)567*, *ΔlacZ4787*(::rrnB-3), λ^-, *rph-1*, *ΔrecG756*, *Δ(rhaD-rhaB)568*, *hsdR514,* [p139]
DA81971	*recJ*	F-, *Δ(araD-araB)567*, *ΔlacZ4787*(::rrnB-3), λ^-, *ΔrecJ743*, *rph-1*, *Δ(rhaD-rhaB)568*, *hsdR514,* [p139]
DA81972	*recN*	F-, *Δ(araD-araB)567*, *ΔlacZ4787*(::rrnB-3), λ^-, *ΔrecN772*, *rph-1*, *Δ(rhaD-rhaB)568*, *hsdR514,* [p139]
DA81973	*recO*	F-, *Δ(araD-araB)567*, *ΔlacZ4787*(::rrnB-3), λ^-, *ΔrecO737*, *rph-1*, *Δ(rhaD-rhaB)568*, *hsdR514,* [p139]
DA81974	*recQ*	F-, *Δ(araD-araB)567*, *ΔlacZ4787*(::rrnB-3), λ^-, *rph-1*, *ΔrecQ767*, *Δ(rhaD-rhaB)568*, *hsdR514,* [p139]
DA81975	*recR*	F-, *Δ(araD-araB)567*, *ΔlacZ4787*(::rrnB-3), *Δ*RecR776:, λ-, rph-1, Δ(rhaD-rhaB)568, hsdR514, [p139]
DA81976	*recT*	F-, *Δ(araD-araB)567*, *ΔlacZ4787*(::rrnB-3), λ^-, *ΔrecT786*, *rph-1*, *Δ(rhaD-rhaB)568*, *hsdR514,* [p139]
DA81977	*recX*	F-, *Δ(araD-araB)567*, *ΔlacZ4787*(::rrnB-3), λ^-, *ΔrecX773*, *rph-1*, *Δ(rhaD-rhaB)568*, *hsdR514,* [p139]
DA81978	*ruvA*	F-, *Δ(araD-araB)567*, *ΔlacZ4787*(::rrnB-3), λ^-, *ΔruvA786*, *rph-1*, *Δ(rhaD-rhaB)568*, *hsdR514,* [p139]
DA81979	*ruvB*	F-, *Δ(araD-araB)567*, *ΔlacZ4787*(::rrnB-3), λ^-, *ΔruvB785*, *rph-1*, *Δ(rhaD-rhaB)568*, *hsdR514,* [p139]
DA81980	*ruvC*	F-, *Δ(araD-araB)567*, *ΔlacZ4787*(::rrnB-3), λ^-, *ΔruvC789*, *rph-1*, *Δ(rhaD-rhaB)568*, *hsdR514,* [p139]
DA81981	*sbcB*	F-, *Δ(araD-araB)567*, *ΔlacZ4787*(::rrnB-3), λ^-, *ΔsbcB780*, *rph-1*, *Δ(rhaD-rhaB)568*, *hsdR514,* [p139]
DA81983	*sbcC*	F-, *Δ(araD-araB)567*, *ΔlacZ4787*(::rrnB-3), *ΔsbcC761*, λ^-, *rph-1*, *Δ(rhaD-rhaB)568*, *hsdR514,* [p139]
DA81982	*sbcD*	F-, *Δ(araD-araB)567*, *ΔlacZ4787*(::rrnB-3), *ΔsbcD762*, λ^-, *rph-1*, *Δ(rhaD-rhaB)568*, *hsdR514,* [p139]
DA81984	Keio *wildtype*	F-, *Δ(araD-araB)567*, *ΔlacZ4787*(::rrnB-3), λ^-, *rph 1*, *Δ(rhaD-rhaB)568*, *hsdR514,* [p139]
DA33135	Chromosome 4.96 Mbp, plasmids 139 kb pDA33135-139, 70 kb pDA33135-70	
DA24990	Isolate carrying the pCP20 plasmid	

Supplementary Table 2. Oligonucleotide primers used for PCR and ddPCR.

Primer name	Sequence (5′→3′)	Target Gene	Application	Amplicon Size (bp)
APH(6)-1d_F<br>APH(6)-1d_R	ATGTTCATGCCGCCTGTTTT<br>TCTGTGCAATGCGTCTAGGA	*aph(6)-1d*	PCR – Verification of the presence of plasmid p139 in Keio strains	580
CTX_M_F<br>CTX_M_R	CCAGTAAAGTTATGGCGGCC<br>CGGTATTGTCGCTGTACTGC	*bla*$_{CTX-M}$	PCR – Verification of the absence of the p70 plasmid in the Keio strains	189
tet(A)_F<br>tet(A)_R	ATCATGGTCCTGCTTGCTTC<br>CTGACGTTCCTCATCCACCT	*tet(A)*	ddPCR – Gene dosage quantification	84
acc(3)-IId_F<br>acc(3)-IId_R	AAACTCCGTTACCGCATTGC<br>ATCTCATACGTCACCCACCG	*acc(3)-IId*	ddPCR – Gene dosage quantification	75
repB_F<br>repB_R	GATAAGTCGTCCGGTGAGCT<br>AGGGTAGGTACAAAGACGCC	*repB*	ddPCR – Plasmid copy number quantification	98
llP_F<br>llP_R	CTACTCTGCTGGCAGGTTGC<br>CACGAGCTGCGTCATCTTTAG	*llp*	ddPCR – chromosomal reference for gene dosage quantification	150

Supplementary Table 3. Tobramycin (TOB) concentrations used for selection of resistant mutants in the PAP assay for individual Keio recombination gene deletions strains carrying the plasmid pDA33135-139. For each recombination gene deletion mutant and the parental wild-type strain, six resistant clones were isolated from two different concentration of TOB *i*: 8×MIC (mg/L) of main population and *ii*: Highest [TOB] supporting subpopulation growth with frequency $\geq 10^{-7}$ (mg/L) from three independent cultures (128 mg/L was the maximum tested concentration).

Strain ID In-house strain collection	Deleted gene	Highest [TOB] not affecting the growth of the main population (mg/L)	[TOB] equivalent to 8×MIC (mg/L)	Highest [TOB] supporting subpopulation growth at frequency $\geq 10^{-7}$ (mg/L)
DA81965	*recA*	0.5	4	16
DA81966	*recB*	0.5	4	16
DA81967	*recC*	1	8	32
DA81968	*recD*	2	16	128
DA81969	*recE*	1	8	128
DA81970	*recG*	0.5	4	32
DA81971	*recJ*	2	16	128
DA81972	*recN*	2	16	128
DA81973	*recO*	1	8	128
DA81974	*recR*	4	32	128
DA81975	*recQ*	1	8	128
DA81976	*recT*	1	8	128
DA81977	*recX*	1	8	64
DA81978	*ruvA*	1	8	64
DA81979	*ruvB*	4	32	128
DA81980	*ruvC*	2	16	32
DA81981	*sbcB*	2	16	128
DA81982	*sbcD*	2	16	128
DA81983	*sbcC*	0.5	4	128
DA81984	Keio wild type	1	8	64

Supplementary Table 4. ddPCR results for the final Keio constructs carrying plasmid pDA33135-139. No changes in gene dosage were detected after the conjugation or at final selection on selective media. The relative ratio was calculated by dividing the ratio of the plasmid genes *aac(3)-IId* and *tet*(A) to the chromosomally located *llp* gene by the average ratio of the same genes in the reference parental HR isolate DA33135. A relative ratio above 2 was considered as indicative of increased gene dosage

DA number of Keio construct	Relative ratio of *acc(3)-IId*/*llp* copy numbers in mutants as compared to DA33135	Relative Ratio of *tet*(A)/*llp* copy numbers in mutants as compared to DA33135
DA81965	1.23	1.04
DA81966	1.57	1.35
DA81967	1.25	1.06
DA81968	1.31	0.99
DA81969	1.21	1.08
DA81970	0.80	0.74
DA81971	1.07	0.92
DA81972	1.02	0.95
DA81973	1.05	0.94
DA81974	1.20	0.92
DA81975	1.13	0.93
DA81976	1.21	1.04
DA81977	1.06	1.03
DA81978	1.22	0.94
DA81979	1.17	0.94
DA81980	1.17	1.08
DA81981	1.09	0.85
DA81982	1.22	0.93
DA81983	1.15	1.13
DA81984	0.93	0.90
DA33135 R1	0.94	0.92
DA33135 R2	1.09	1.09
DA33135 R3	0.99	1.00
DA33135 R4	0.98	0.98

Supplementary Table 5. Whole-genome sequencing results for recombination gene deleted mutants carrying plasmid pDA33135-139. Gene names in bold indicate genes known to confer aminoglycoside resistance when mutated in various bacterial species. Genetic alterations identified for each gene are shown in parentheses. “None” indicates that no genetic changes were detected in the mutant based on the WGS analysis. “*” denotes a stop codon, and “nt” denotes nucleotide(s).

Strains DA number	Genetic changes	Possible mechanism behind aminoglycoside resistance	reference
Knocked-out gene: *recA*			
DA83149	*lpcA* (L116Q), *repB* (S36P), ***rpsL*** (H77L)	RpsL (ribosomal protein) mutations (commonly mutations at K43R and K88Q) lead to aminoglycoside resistance by altering the binding of the antibiotic.	(1)
DA83342	*lpcA* (L116Q), *pepP* (A25fs), ***fusA*** (M461Thr)	FusA (elongation factor) mutations might change the ribosomal structure and interfere with drug binding.	(2,3)
DA83344	*lpcA* (L116Q), ***kdpD*** (E110G), *ydaU* (D163N)	KdpD (two-component system KdpDE) mutations might change the membrane potential leading to reduced aminglycoside uptake.	(4)
DA83345	*lpcA* (L116Q), *fre* (R178fs)		
DA83521	*lpcA* (L116Q), ***ubiH*** (D325fs), *kdpD* (D460V)	UbiH (ubiquinone biosynthesis) mutations lead to a decreased proton-motive force across the bacterial membrane, which reduces the uptake of aminoglycoside antibiotics.	(4,5)
Knocked-out gene: *recB*			
DA83150	*ykgE* (A132E), ***ubiF*** (H294fs), *ptrA* (P955L)	UbiF (ubiquinone biosynthesis) mutations lead to a reduction in proton motive force leading to reduced aminoglycoside uptake.	(6)
DA83346	*ykgE* (A132E), *ptrA* (P955L)		
DA83347	*ykgE* (A132E), *ptrA* (P955L)		
DA83348	*ykgE* (A132E), *aceE* (H408L)		
Knocked-out gene: *recC*			
DA83352	***hemA*** (L112Q)	HemA (biosynthesis of heme) mutations lead to reduced aminoglycoside uptake.	(7)
DA83353	***hemA*** (L112Q)	HemA (biosynthesis of heme) mutations lead to reduced aminoglycoside uptake.	(7)
Knocked-out gene: *recD*			

DA83158	None		
DA83363	None		
DA83364	*bax* (K46Q)		
DA83365	*bax* (K46Q)		
Knocked-out gene: *recG*			
DA83379	None		
Knocked-out gene: *recJ*			
DA83170	***cyoC*** (Ins 4 nt A87fs), ylbE (A332E)	CyoC (cytochrome terminal oxidase complex) mutations might lead to a reduction in proton motive force leading to reduced aminoglycoside uptake.	(8,9)
DA83388	***cyoC*** (Ins 4 nt A87fs), ylbE (A332E)		(8,9)
Knocked-out gene: *recN*			
DA83175	*moaA* (K270*)		
DA83395	*moaA* (K270*)		
DA83397	*moaA* (K270*)		
Knocked-out gene: *recQ*			
DA83420	***hemC*** (V85A), *fdnG* (V611G), *dinB* (IS26 transposone gene insertion)	HemC (biosynthesis of heme) mutations might lead to reduced aminoglycoside uptake.	(7)
Knocked-out gene: *recX*			
DA83194	None		
Knocked-out gene: *ruvC*			
DA83456	***fusA*** (Q129K), *ribE* (F2L), *lrhA* (L118*)	FusA (elongation factor) mutations might change ribosome structure and interfere with drug binding.	(2,3)
Knocked-out gene: *sbcB*			
DA83462	***hemA*** (P115Q)	HemA (biosynthesis of heme) mutations lead to reduced aminoglycoside uptake.	(2)
Keio wild-type			
DA83222	none		
DA83493	none		

References

1. Ghosh A, Saran N, Saha S. Survey of drug resistance associated gene mutations in Mycobacterium tuberculosis, ESKAPE and other bacterial species. Sci Rep. 2020;10(1):8957.
2. Buckel P, Buchberger A, Böck A, Wittmann HG. Alteration of ribosomal protein L6 in mutants of Escherichia coli resistant to gentamicin. Mol Gen Genet. 1977;158(1):47–54.
3. Dongping W, J DR, S HC, Chien-chi L, K DAE, S CP, et al. Adaptation Genomics of a Small-Colony Variant in a Pseudomonas chlororaphis 30-84 Biofilm. Appl Environ Microbiol. 2015 Feb 1;81(3):890–9.
4. Wang H, Lu H, Jiang C, Zhu L, Lu H. High-Throughput Evolution Unravels Landscapes of High-Level Antibiotic Resistance Induced by Low-Level Antibiotic Exposure. 2023;
5. Rodrigo AC, Katayoun KF, Emmanuel S, Ludovic P, Corinne L, Fabien P, et al. Role of the Escherichia coli ubiquinone-synthesizing UbiUVT pathway in adaptation to changing respiratory conditions. mBio. 2023;14(4):e03298-22.
6. Ma C, Sim S, Shi W, Du L, Xing D, Zhang Y. Energy production genes sucB and ubiF are involved in persister survival and tolerance to multiple antibiotics and stresses in Escherichia coli. FEMS Microbiol Lett. 2010;303(1):33–40.
7. Kirst HA, Allen NE. Aminoglycosides antibiotics. Comprehensive Medicinal Chemistry II. 2006;7:629–52.
8. Suzuki S, Horinouchi T, Furusawa C. Prediction of antibiotic resistance by gene expression profiles. Nat Commun. 2014;5:5792.
9. Charron R, Lemée P, Huguet A, Minlong O, Boulanger M, Houée P, et al. Polyhexamethylene biguanide promotes adaptive cross-resistance to gentamicin in Escherichia coli biofilms. Front Cell Infect Microbiol. 2023;13:1324991.
